# Impact of Emerging Antiviral Drug Resistance on Influenza Containment and Spread: Influence of Subclinical Infection and Strategic Use of a Stockpile Containing One or Two Drugs

**DOI:** 10.1371/journal.pone.0002362

**Published:** 2008-06-04

**Authors:** James M. McCaw, James G. Wood, Christopher T. McCaw, Jodie McVernon

**Affiliations:** 1 Vaccine and Immunisation Research Group, Murdoch Childrens Research Institute and Melbourne School of Population Health, The University of Melbourne, Parkville, Victoria, Australia; 2 School of Public Health and Community Medicine, University of New South Wales, Sydney, New South Wales, Australia; 3 National Centre for Immunisation Research and Surveillance of Vaccine Preventable Diseases, The Childrens Hospital at Westmead and the University of Sydney, Sydney, New South Wales, Australia; U.S. Naval Medical Research Center Detachment/Centers for Disease Control, United States of America

## Abstract

**Background:**

Wide-scale use of antiviral agents in the event of an influenza pandemic is likely to promote the emergence of drug resistance, with potentially deleterious effects for outbreak control. We explored factors promoting resistance within a dynamic infection model, and considered ways in which one or two drugs might be distributed to delay the spread of resistant strains or mitigate their impact.

**Methods and Findings:**

We have previously developed a novel deterministic model of influenza transmission that simulates treatment and targeted contact prophylaxis, using a limited stockpile of antiviral agents. This model was extended to incorporate subclinical infections, and the emergence of resistant virus strains under the selective pressure imposed by various uses of one or two antiviral agents. For a fixed clinical attack rate, *R*_0_ rises with the proportion of subclinical infections thus reducing the number of infections amenable to treatment or prophylaxis. In consequence, outbreak control is more difficult, but emergence of drug resistance is relatively uncommon. Where an epidemic may be constrained by use of a single antiviral agent, strategies that combine treatment and prophylaxis are most effective at controlling transmission, at the cost of facilitating the spread of resistant viruses. If two drugs are available, using one drug for treatment and the other for prophylaxis is more effective at preventing propagation of mutant strains than either random allocation or drug cycling strategies. Our model is relatively straightforward, and of necessity makes a number of simplifying assumptions. Our results are, however, consistent with the wider body of work in this area and are able to place related research in context while extending the analysis of resistance emergence and optimal drug use within the constraints of a finite drug stockpile.

**Conclusions:**

Combined treatment and prophylaxis represents optimal use of antiviral agents to control transmission, at the cost of drug resistance. Where two drugs are available, allocating different drugs to cases and contacts is likely to be most effective at constraining resistance emergence in a pandemic scenario.

## Introduction

Concerns regarding emergence of a pandemic influenza strain have prompted the development of national and international preparedness plans by governments and public health agencies. As a key element of these plans, many developed nations have acquired large stockpiles of antiviral drugs to be used for treatment of infected individuals and for the containment and mitigation of virus transmission. Antivirals may be deployed to curtail an epidemic for sufficient time to allow development and deployment of a targeted vaccine. In previous work, we developed a simple, analytically tractable mathematical model to explore the likely relative effects on epidemic dynamics of treatment and targeted prophylaxis using a finite stockpile of such agents [1]. In keeping with studies using computationally intensive individual-based models, we concluded that, for the purposes of limiting spread, prevention was better than cure [2], [3].

Widespread use of antiviral drugs, however, has the potential to promote emergence of resistant strains. The consistent conclusion from models examining this issue [4] [5] [6] [7] [8] [9] [10] is that relative fitness of mutant viruses is the key characteristic determining their influence on epidemic dynamics and hence antiviral effectiveness. Given that a high fitness cost has been observed among most neuraminidase-inhibitor resistant influenza strains to date [11] [12], this finding provides some reassurance for health policy planners.

The effect of differing patterns of drug use on emergence of resistant viruses is more contentious. Depending on a range of underlying assumptions including the characterisation of asymptomatic individuals and population size, either treatment [4] [10] or prophylaxis-based [5] [7] strategies were predicted as more likely to select for resistant strains. In addition to incorporating the likely development of resistance, we have explored in detail the importance of infections attributed to asymptomatic individuals in a large-scale population of 20 million people. We have further considered how the strategic use of a finite stockpile containing two antiviral drugs might minimise the emergence of resistance or mitigate its negative effects during a pandemic, and suggest that using separate drugs for treatment and prophylaxis might be an optimal strategy, where feasible.

## Methods

### The basic model

The susceptible-exposed-infectious-recovered (SEIR)-based model of influenza transmission has been described in detail elsewhere [1]. A novel feature is the incorporation of a dynamic ‘contact’ label, applied to a fixed number of individuals drawn from the whole population each time a new infectious case appears. We define these contacts, based on the findings of sociological studies, as those people who have been sufficiently close to an infected individual to conceivably contract infection [13] [14] [15], and so may be considered eligible for prophylaxis. Infections can only occur in individuals who have been in contact with a case, with the uninfected contacts, who comprise the majority, returning to their original ‘non-contact’ status within a matter of days.

### Infectious duration

Considerable uncertainty exists regarding the maximal infectiousness of individuals in relation to the symptomatic course of influenza, although viral shedding data has been related to infectious potential [16] [17]. We do not explicitly characterise symptom onset in our model, but assume a one-day latent period (*E*) between inoculation and the onset of three days' constant infectious duration (*I*), exponentially distributed over the period, of which at least part of one day is likely to be presymptomatic.

### Incorporation of asymptomatic infection

**Figure 1:**
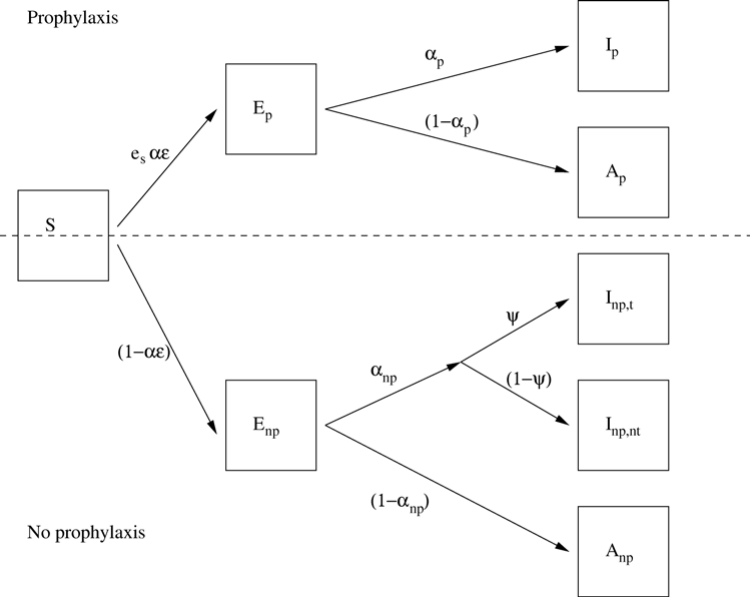
Model schematic. Simplified schematic of the model in the case of no resistance. In the steady state a proportion, ε, of contacts of symptomatic cases who are susceptible will have received prophylaxis (*p* subscript, above the dotted line). The remainder have not (*np* subscript, below the dotted line). Those on prophylaxis have a reduced susceptibility *e_s_*. For details refer to [1]. A proportion, *ψ*, of symptomatic infections that were not provided with prophylaxis receive treatment. For clarity, we have not shown the progression from the infectious (*I*, *A*) states to the permanently recovered (*R*) states. The force of infection arises from the five states on the right. Relative to the *I_np_*_,*nt*_ state, the infectiousness of the *I_p_*, *A_p_*, *I_np_*_,*t*_ and *A_np_* states are reduced by the factors *e_i_*, *e_i_χ*, *e_t_* and *χ* respectively.

Comparison of disease and seroconversion rates in studies of seasonal influenza demonstrates a high proportion of subclinical episodes [18]. Historical cohort studies of influenza have associated asymptomatic seroconversion with pre-season immunity, presumably derived from past infection with related viruses [19]. Even for novel pandemic strains, evidence of long-lived protection resulting from exposure to conserved viral antigens is demonstrated by differential age-specific attack rates and relative sparing of older age cohorts in historical reports [20] [21] [22]. Modelling studies of past pandemics suggest asymptomatic infection may have accounted for one third to half of all infections [23] [24]. To date, little evidence of asymptomatic seroconversion has been demonstrated among close contacts of individuals infected with H5N1 avian influenza [25] [26], but whether this feature would be shared by a strain with pandemic potential cannot be predicted. We therefore explore the sensitivity of model behaviour to a range of assumptions regarding the symptomatic proportion (*α*) in the range (*x*,1) for a given clinical attack rate *x*. Asymptomatic cases cannot be identified and treated, nor can their contacts be provided with prophylaxis. Figure 1 shows a schematic of the basic model structure.

While it appears reasonable to assume that asymptomatic individuals are less infectious than those with rhinorrhea, sneezing and coughing, ‘off-season’ transmission of influenza within households has been reported, without illness [19]. The absence of data from which to estimate the relative infectiousness of subclinical influenza is apparent from published models, in which parameter assignments range anywhere from zero [7] to one tenth [4] [8], to effectively double [5] that of clinical infections. We approach this issue by investigating the impact of the relative infectiousness of subclinical infections (*χ*) in the range (0,1) on model dynamics and the effectiveness of interventions.

### Incorporation of resistant strains – characterisation of behaviour relative to wild type

The emergence of antiviral-resistant strains is characterised by a ‘seeding’ parameter (*ρ*) explored across the range (10^−4^, 10^−1^). This parameter describes the proportion of hosts initially infected with wild-type virus who, as a result of within-host selective pressure exerted by antiviral agents, become predominantly infected with, and transmit, resistant viruses. As earlier initiation of drug therapy is likely to result in greater suppression of viral growth, we assume with others [6], [7] that the probability of seeding resistance by prophylaxis (*ρ_p_*) is 10-fold smaller than by treatment (*ρ_t_*). The transmission fitness of mutant strains relative to wild type is described by an additional parameter (*φ*). We assume that asymptomatic infection occurs in equal proportions for resistant and wild type strains, as we propose that this fraction is primarily influenced by the distribution of prior immunity in the host population resulting from past virus exposure. Asymptomatic infections for both wild-type and resistant strains are assumed to be χ times as transmissible as symptomatic infections.

The model allows distinct parameterisation of resistant strain seeding rates and fitness under selective pressure imposed by two antiviral drugs. Given high rates of spontaneous resistance of influenza virus strains against the adamantane class, the modelled drugs may be considered as the neuraminidase inhibitors (NAIs) oseltamivir and zanamivir. While there are fewer case reports of zanamivir [27] than oseltamivir resistance in the literature, insufficient data exist to inform the likely resistance profile of influenza strains following widespread use of these agents. In the absence of such data, we have assigned equivalent seeding and fitness parameters for both strains in the present study.

### Strategic drug use to minimise emergence/impact of resistance

Targeted antiviral prophylaxis may be distributed to a proportion (*ε*) of contacts of symptomatic infectives, reducing susceptibility to infection (*e_s_*) [28] [29]. Where breakthrough infection occurs, reduced infectiousness is assumed (*e_i_*), consistent with the finding of marked reduction in influenza virus shedding with prompt antiviral therapy [30]. Providing treatment within 48 hours of symptom onset to a proportion (*ψ*) of cases arising in the absence of prophylaxis also reduces infectiousness (*e_t_*) [28] [29].

We compare plausible ways in which one or two finite NAI drug stockpiles could be deployed for treatment and prophylaxis in a pandemic scenario. Two summary measures are used to compare the effectiveness of alternative strategies: (a) Emergence of resistance is measured using the cumulative proportion of all strains resistant to one or both drugs, as a function of time (b) The impact of mutant strains on outbreak dynamics is assessed by the time to reach half of the final attack rate (*t*_med_). The latter measure is more robust than time to epidemic peak, when multiple peaks are observed.

We begin with an outline of the most effective use of a single drug by comparing effects of treatment of symptomatic cases, prophylaxis of close contacts and a combination of both. We then extend this analysis to the optimal allocation of a stockpile containing two drugs (in variable proportions) within a combined treatment and prophylaxis strategy as follows:

(i) random distribution, limited only by the proportion of each drug remaining in the stockpile at any point in time,
(ii) periodic drug cycling over a range of frequencies,
(iii) use of one drug for treatment only and the other for prophylaxis of close contacts.

Under all strategies, the total number of antiviral courses available is fixed at 44% of the population size, consistent with per capita estimates of the Australian antiviral stockpile at the end of 2006.

Strains resistant to one drug are assumed to be sensitive to the alternative agent, although sequential development of multi-drug resistance may occur. We do not allow for reversion of resistant strains to wild type in the absence of ongoing selective pressure from antiviral drugs. Modifications to the one-drug model required to implement these strategies are described in detail in Appendix S1.

## Results

### Asymptomatic infection

**Figure 2:**
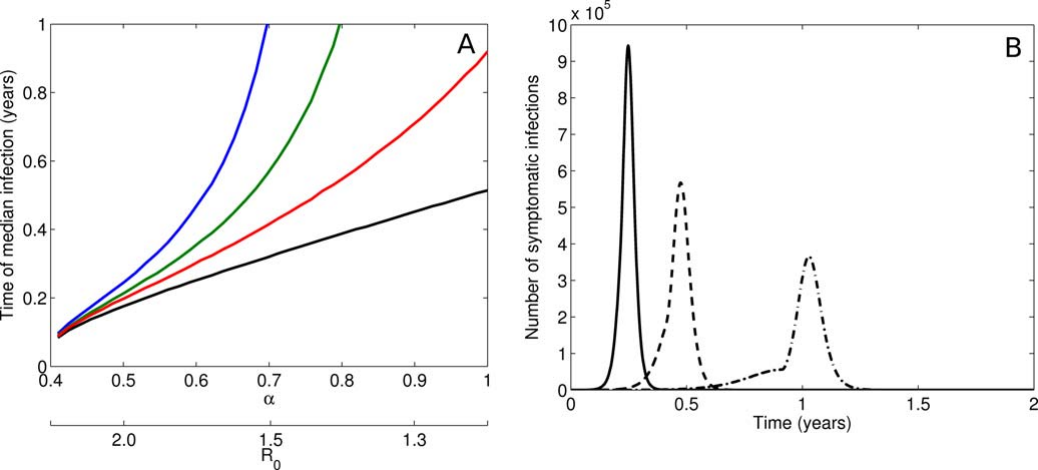
The influence of asymptomatic infection. Time of median infection as a function of the symptomatic proportion for a fixed clinical attack rate of 40%. No intervention (black (*ψ* = 0.0, *ε* = 0.0)), treatment only (red (*ψ* = 0.4, *ε* = 0.0)), prophylaxis only (green (*ψ* = 0.0, *ε* = 0.3)) and combined treatment and prophylaxis (blue (*ψ* = 0.4, *ε* = 0.3)). b. Epidemic curves for the combined intervention case in Figure 2a, for *α* = 0.5 (solid), *α* = 0.6 (dashed) and *α* = 0.7 (dot-dashed). For a severe epidemic (low *α*, high *R_0_*) the intervention is ineffective. For a mild epidemic (high *α*, low *R_0_*) the intervention prevents most transmission until the stockpile expires at which point the epidemic takes off quickly. Stockpile expiry occurs at the kink in the epidemic curve for *α* = 0.7.

Figure 2a) demonstrates the relationship between *α* and *R_0_* in the absence of drug resistance. For a fixed clinical attack rate of 40%, a decrease in the proportion of symptomatic infections (ie lower *α*) is consistent with a greater reproductive number and serological attack rate. Consequently, both the time from the first observed case to *t*_med_ and the delay induced by interventions are reduced. Consistent with our earlier work [1], treating 40% of symptomatic cases (*ψ* = 0.4) has less effect on the spread of infection than providing prophylaxis to 30% of their contacts (*ε* = 0.3), and strategies that combine the two approaches are optimal, across the full range of *α*. The change in effectiveness of a combined intervention as the asymptomatic proportion varies is demonstrated in Figure 2b). For a 50% asymptomatic infection rate, the epidemic is largely uncontrolled. As the asymptomatic fraction falls, transmission is constrained until stockpile expiry, at which point an explosive outbreak occurs.

### Resistant virus behaviour

**Figure 3:**
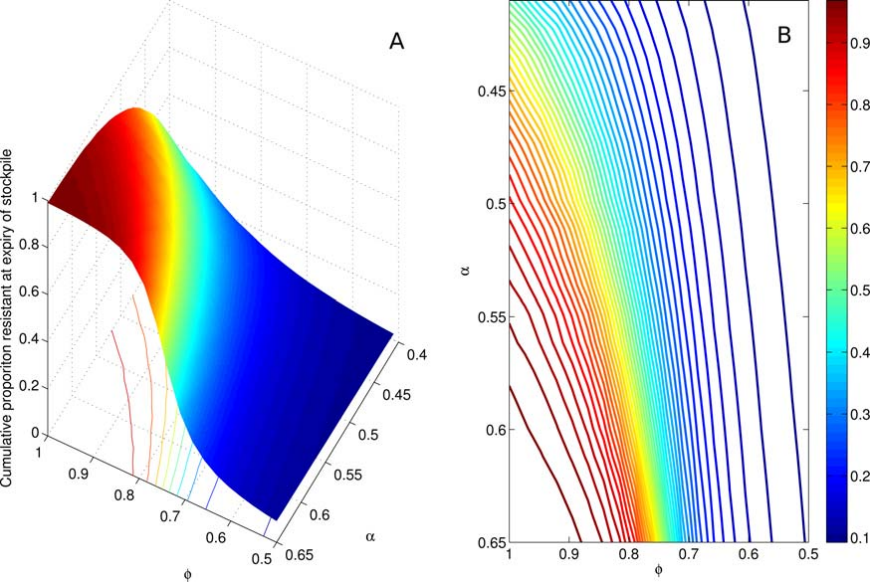
The proportion of cumulative infections that is resistant. a. Surface plot of the proportion of the total cumulative infections that are resistant, at the point of stockpile expiry. The transition (“cliff-edge”) from a low-transmissible to high-transmissible resistant strain occurs at *φ*≈0.7. As α increases (*R_0_* decreases) the wild-type strain is less transmissible, the intervention is more effective, and thus the resistant strain is more capable of dominating transmission. b. Contour plot as for Figure 3a.

We now allow for the possibility of emergence of a resistant virus strain arising at a relatively high rate (*ρ*_t_ = 10^−1^, *ρ*_p_ = 10^−2^) in the presence of a single antiviral drug used according to the combined treatment (40%) and prophylaxis (30%) strategy. The transmissibility, *φ*, of this strain relative to the uncontrolled wild type virus is a key driver of the cumulative proportion of all infections that are resistant, measured at the time of stockpile expiry. A clear threshold effect is observed as a ‘cliff-edge’ in the region of 70–90% relative transmissibility in the surface and contour plots (Figures 3a and b). Resistance is further influenced by the asymptomatic proportion, becoming more prevalent for higher assumed values of *α* where a larger fraction of infections are clinically observed, prompting drug distribution for treatment and prophylaxis. Moreover, given the lower baseline R_0_, interventions produce longer delays to *t*_med_, allowing more time for propagation of resistant strains before drug stockpile expiry.

**Figure 4:**
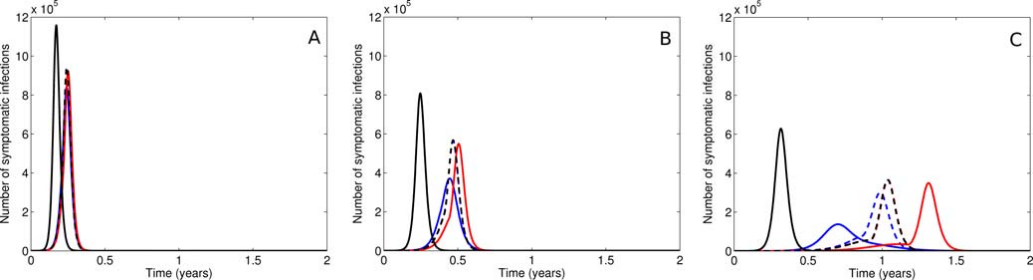
The impact of seeding and fitness on viral dynamics. Epidemic curves. No intervention (solid black), no resistance (dashed black), high fitness (blue) with high seeding (solid) and low seeding (dashed) and low fitness (red) with high seeding (solid) and low seeding (dashed). a. Epidemic curves for high/low seeding and high/low fitness and *α* = 0.5. The intervention has a marginal effect for all combinations of resistant virus strain properties. b. Epidemic curves for high/low seeding and high/low fitness and *α* = 0.6. The intervention has a small effect. High fitness resistant strains (blue) result in a marginally shorter time to epidemic peak than in the case of a low fitness resistant strain or no emergence of resistant strains. c. Epidemic curves for high/low seeding and high/low fitness and *α* = 0.7. The intervention has a significant impact on the dynamics. For high fitness (blue), the resistant strain can dominate and dramatically lessen the time to epidemic peak. For low fitness, the epidemic is well controlled until stockpile expiry. A high seeding rate (solid red) provides an “immunising effect” which results in a dramatic delay in time to median infection.

The combined influence of the rate of resistance emergence and the relative fitness of mutant strains on epidemic dynamics is demonstrated in Figure 4. Graphs a), b) and c) depict outbreak curves for assumed values of the symptomatic proportion of 50, 60 and 70% respectively. In each of these scenarios, epidemic curves are shown at baseline (no intervention), and with a combined treatment (40%) and prophylaxis (30%) strategy in the absence of antiviral resistance. Four possible combinations of resistant virus characteristics are then explored in the intervention case – high transmissibility (*φ* = 0.8) with high or low seeding, and low transmissibility (*φ* = 0.3) with high or low seeding.

As before, a low symptomatic infection rate (50%) results in an essentially uncontrolled epidemic at this level of intervention. With an increase in the symptomatic proportion, greater delays are achievable, and the properties of the resistant strain begin to have a sizeable effect on timing. As Figure 4c) demonstrates, a ‘fit’ mutant arising at a high incidence rate dominates transmission relative to the controlled wild-type virus, resulting in an earlier onset but less explosive epidemic than observed in the absence of resistance. Conversely, an unfit strain with a high seeding rate is unable to propagate, effectively ‘immunising’ the population and thus further delaying the wild type outbreak associated with stockpile expiry.

### Strategic use of two drugs

We now consider ways in which two NAIs with distinct resistance profiles may be deployed in order to mitigate the deleterious effects of a high fitness/high seeding resistant strain on outbreak control. For continuity, we continue to deliver a combined treatment (40%) and prophylaxis (30%) regimen. A symptomatic fraction of 70% is assumed, as this allows the effects of different interventions to be most clearly demonstrated. The relative proportions of drugs in the stockpile are set at either 90/10% or 50/50%.

#### Strategy 1: Random allocation

**Figure 5:**
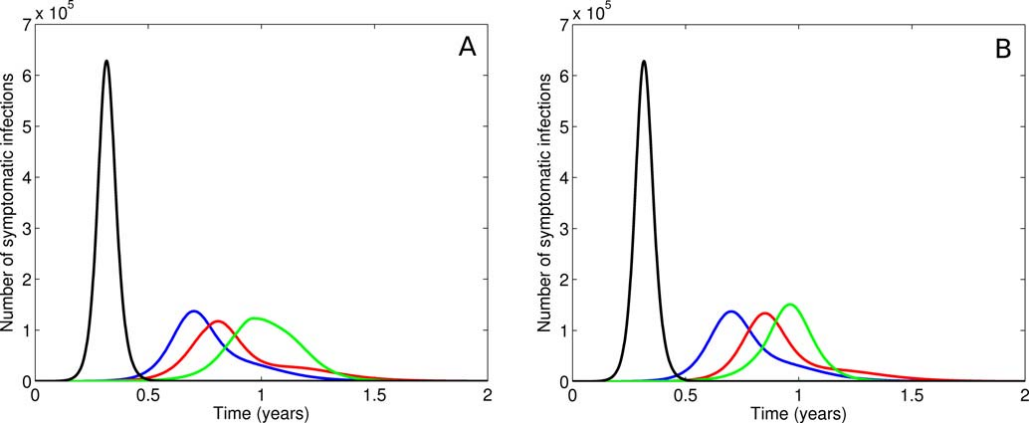
Strategic use of one or two drugs: epidemic curves. a. Epidemic curves for no intervention (black), a single drug strategy (blue), a random allocation strategy (red) and a T&P strategy (green) for a 90/10 stockpile. The T&P strategy provides the longest time to median infection. b. As for Figure 5a but with a 50/50 stockpile.

**Figure 6:**
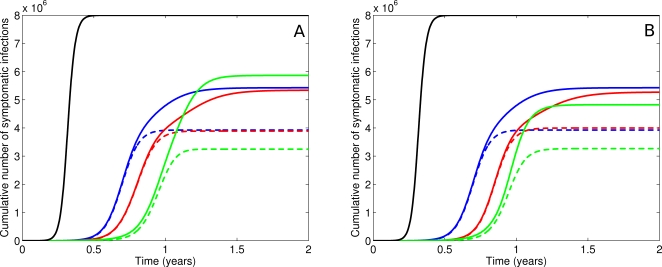
Strategic use of one or two drugs: cumulative infections. a. Cumulative infections for a 90/10 stockpile. Colours as in Figure 5a. The solid line is total infections. The dotted line is resistant infections (single-drug and multi-drug resistant). All interventions result in a reduced attack rate. The T&P strategy has a measurably reduced proportion of resistant infections and thus, at stockpile expiry, the wild-type strain dominates, resulting in the highest overall attack rate (but the longest delay). b. As for Figure 6a but with a 50/50 stockpile.

**Figure 7:**
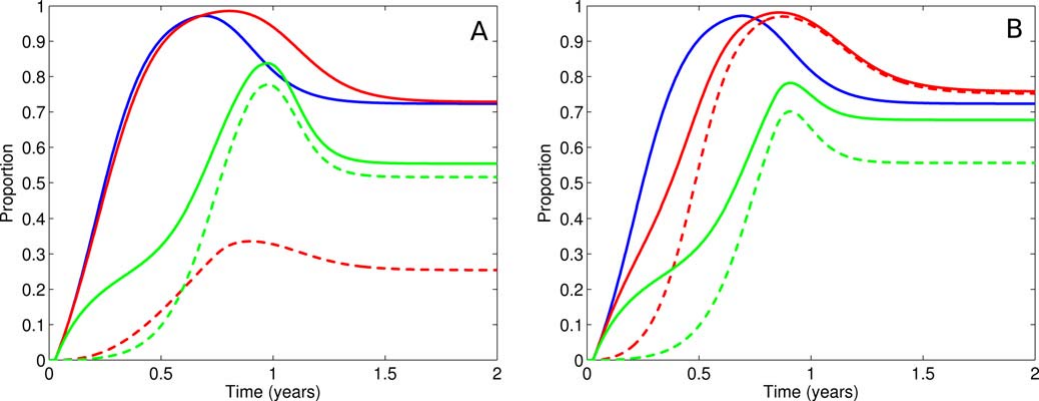
Strategic use of one or two drugs: the resistant proportion. a. Proportion of cumulative infections that are resistant for a 90/10 stockpile. Colours as in Figure 5a. Solid lines are all resistant strains (single-drug and multi-drug resistant). Dashed lines are multi-drug resistant strains only. Random allocation (red) is dominated by single-drug strain resistance. T&P (green) is dominated by multi-drug strain resistance, but as a proportion of all infections, there is less resistance overall. b. As for Figure 7a but for a 50/50 stockpile. Both random allocation (red) and T&P strategies (green) are dominated by multi-drug strain resistance. The T&P strategy has less resistance overall.

Drugs are randomly distributed for either treatment or prophylaxis, as indicated, depending on the proportions of Drug 1 and 2 in the stockpile. With a 90/10% split, longer delays to *t*_med_ are achieved than in the one drug scenario (Figure 5a). Resistance to the drug in greater supply dominates the epidemic (Figure 6a) and multi-drug resistance is rare (Figure 7a). Where relative drug proportions are changed to 50/50%, a marginal increase in time to outbreak is achieved (Figure 5b), at the cost of more multi-drug resistance (Figure 7b).

#### Strategy 2: Drug cycling

**Figure 8:**
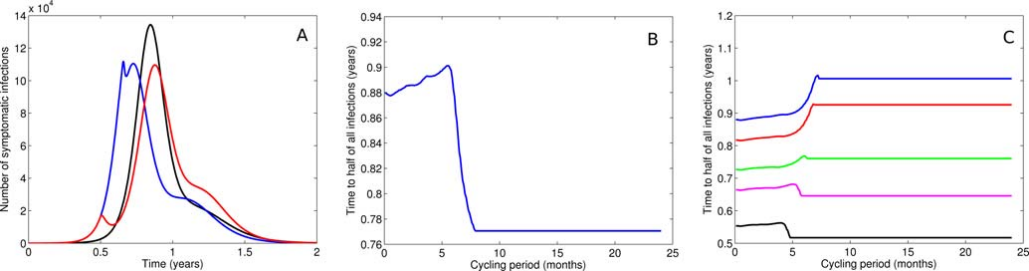
Analysis of the two drug cycling strategy. a. Epidemic curves with a 90/10 stockpile, for random allocation (black), using the 90% stockpile first (blue) and using the 10% stockpile first (red). Using the 90% stockpile first results in a poor outcome. Using the 10% stockpile first results in a slightly improved outcome compared to a random allocation strategy. b. Time to median infection (*t*_med_) vs cycling period for a 90/10 stockpile. The 90% drug is used first. If the switch is not made soon enough, the time to median infection drops below the time in a random allocation strategy. c. Time to median infection (*t*_med_) vs cycling period for a 50/50 stockpile. From top to bottom, the fitness of the resistant strain(s) is subtly increasing (*φ* = 0.8, 0.81, 0.83, 0.85, 0.9). As the cycling period increases, the delay increases until a threshold is reached. Beyond the threshold the time to median infection may be greater than or less than the time for a random allocation strategy (cycling period approaching zero).

Individual drugs are deployed in the population over weeks or months, generally achieving greater delays to *t*_med_ than in Strategy 1. Given the assumption of no cross-resistance, a sizeable reduction in transmission occurs when Drug 2 is introduced in a population with a high prevalence of resistance to Drug 1. As only one drug is in use for either treatment or prophylaxis at any point in time, multi-drug resistance cannot develop in this scenario before the second antiviral agent is distributed. It should be noted, however, that the periodic perturbations induced by drug switching may result in highly complex behaviour, with unpredictable and frequently unfavourable consequences for outbreak control. Such outputs are examined in detail in the Appendix S1 – key summary points are as follows:

1. Where cycle length is sufficiently long to allow depletion of one or other drug before switching and the stockpile is asymmetric, the drug in shorter supply should be used first. This is because the lesser quantity is unlikely to last long in the later phases of an exponentially growing epidemic (Figure 8a).
2. In the case of the 90/10% stockpile, increasing the cycle length generally delays the time to half the final attack rate as multi-drug resistance cannot emerge until the second drug is used. However, if the length of the first drug cycle is too great, single-drug resistance reaches such high prevalence that therapeutic efficacy declines (Figure 8b). Such wastage of the finite stockpile ultimately results in less effective containment than Strategy 1.
3. Perturbations induced by drug switching may in some instances result in a substantially shorter time to the median case than random drug allocation. The exquisite sensitivity of this behaviour to unknown (and unmeasurable) parameter assignments is demonstrated by Figure 8c) which plots the time to half the final attack rate against cycling period using a 50/50% stockpile for resistant virus strains of variable fitness. Divergent effects of cycling time are observed for subtly different transmission parameter assignments (Range *φ = *0.8, 0.9).

#### Strategy 3: Separate drugs for prophylaxis and treatment

Results from the single drug combined intervention strategy demonstrate that more than 90% of the stockpile is deployed for prophylaxis, and the remainder for treatment (data not shown). The allocation of a 90/10% drug stockpile is thus limited to using the drug in greater supply for prophylaxis and the alternative for treatment. Onset of drug resistance occurs later than in other scenarios (Figure 6a), but is dominated by multi-drug resistant strains (Figure 7a). In consequence, this strategy provides the longest achievable delays to *t*_med_ of all interventions explored (Figure 5a).

When the stockpile is symmetric (50/50%), the absolute number of antiviral doses available for prophylaxis is substantially reduced. However, no apparent difference in resistance emergence (Figure 6b), 7b)) or epidemic timing (Figure 5b)) results from this reduced supply for prophylaxis. As we have previously demonstrated, drug delivery mirrors epidemic growth, which is exponential [1]. It follows that, in the absence of logistic constraints, a substantial proportion of the stockpile will be distributed in a relatively short timeframe immediately prior to depletion. Conversely, doubling the stockpile may control the epidemic for only a few days more, not twice as long. For the 50/50% scenario, a proportion of the treatment stockpile remains unused at the end of the epidemic.

### Sensitivity analysis

Only the key results are described here: an extensive sensitivity analysis of relevant parameters characterising the virus and interventions is provided in Appendix S1. It is worth noting that, for the two drug models, qualitative conclusions regarding the relative benefits of alternative strategies might change where the assumption of equivalent drug efficacy is allowed to vary.

## Discussion

Our results demonstrate that the asymptomatic proportion of infections is a critical determinant of the ability to constrain an outbreak with a fixed level of interventions. When the epidemic is controllable, antiviral distribution strategies that combine treatment and prophylaxis result in the greatest reduction in virus transmission, with the longest achievable delays to the median case. In the context of ‘buying time’ for development and deployment of a targeted vaccine, this is a desirable strategy. The cost from this combined intervention is that it also leads to the highest rates of resistant infections. This is not surprising, as treatment favours the emergence of resistance in our model, while prophylaxis provides selective advantage for propagation of mutant strains in the host population.

This synergistic promotion of resistance can be curtailed by the provision of different antiviral drugs with distinct resistance profiles. In particular, if separate drugs are used for treatment and prophylaxis, the chain of transmission of resistant viruses is broken, prolonging effectiveness of the intervention. While cycling strategies delay the emergence of multi-drug resistance, and may be advantageous in some instances, benefit cannot be consistently predicted.

A particular strength of our approach is the use of dynamic ‘contact’ variables [1], which enable simulation of targeted drug distribution and stockpile depletion in a large population. In consequence, we can consider the implications of one or two finite stockpiles expiring, which include resurgence of wild-type infections following drug depletion, with consequences for the final cumulative proportion of all infections that are resistant.

It should be noted that our use of a deterministic model has the usual limitations. The inherently stochastic nature of the epidemic in its early stages, and the initial seeding of a drug resistant strain, cannot be accounted for accurately. The dynamics of the established epidemic however should be well captured.

The characterisation of resistance in the model was subject to several simplifying assumptions. Firstly, we assumed that resistance arose only within human hosts receiving antiviral therapy. Recent surveillance for neuraminidase inhibitor resistance in influenza viruses has detected spontaneous mutations conferring reduced drug sensitivity, in isolates from regions where use of antiviral agents is rare [31] [32] [33]. Evidence of oseltamivir persistence in treated waste-water and the prophylactic use of antiviral agents in poultry production also raises the potential for resistance selection in avian populations, prior to introduction to human hosts [34]. These observations could invalidate our baseline assumption of fully sensitive strains.

We further assume that a host who becomes infected with a resistant virus will continue to propagate such a strain, even if they do not receive antiviral drugs themselves. While within-host reversion to wild-type may well occur in the absence of selective pressure, this becomes less likely for strains with little biological impairment [35]. On balance, we therefore felt it most conservative to disregard this possibility in order to explore the worst-case scenario of rapid development of multi-drug resistance. If resistance to one drug also conferred resistance to the other, our two-drug model would effectively reduce to the single-drug case.

The transmission potential of an emergent pandemic influenza strain is subject to debate. Even more uncertain is the likely rate of emergence and fitness of resistant strains in the setting of wide-scale use of antiviral agents. Resistant mutants, where reported in animal experiments, have generally exhibited markedly reduced transmissibility [36]. Mutant viruses have been detected in up to 18% of paediatric subjects treated with oseltamivir in clinical trials [37]. The low prevalence of oseltamivir resistance in Japan [38], where this drug has been used extensively, has provided some reassurance that such strains are not well propagated. However, more recent NAI resistance surveillance data from the United States [32] and Europe [33] have highlighted the emergence of mutants that appear to be readily transmissible between humans. The H274Y mutation, identified in 14% of 437 European strains (70% of Norwegian strains) between November 2007 and January 2008, is associated with a 400-fold reduction in susceptibility to oseltamivir. None of these mutant isolates appear to have been taken from treated individuals, making it most likely that they are readily transmissible between humans [39]. Given such wide diversity of observations, we have therefore aimed to characterise the full spectrum of behaviours of wild type and resistant virus that might be observed within a flexible model framework.

In the main text, we have concentrated on a few key examples but discuss a wider range of scenarios in Appendix S1. For example, we show that more rapidly growing epidemics (those with a higher asymptomatic proportion) may be well controlled by applying increased levels of intervention, within the limits of feasibility. We have deliberately chosen more modest levels of drug distribution, which might be achievable in a public health emergency. Further, we show that strains with higher transmissibility may be constrained if a corresponding reduction in seeding rate is assigned. The relative advantage of alternative drug strategies is not altered by changes to these assumptions.

Variation in strain behaviour could also influence the efficacy of antiviral agents at the individual host level. Recent work in ferrets has shown marked differences in the virulence potential of H5N1 influenza viruses arising from distinct clades. Earlier treatment onset, higher daily dosages and a longer course of oseltamivir were required to prevent morbidity and mortality among ferrets infected with the more virulent strain [40]. Inadequate dosing is also more likely to favour the emergence of drug resistance [40], but as the optimal recommendations and compliance in a pandemic situation are purely speculative at present, we have not attempted to characterise this phenomenon within the model. Population heterogeneity, which we have not incorporated, could impact on antiviral interventions in several ways. For example, a recent study has demonstrated more persistent virus shedding 4–6 days after initiation of oseltamivir therapy among children than adults [41], consistent with earlier findings [37]. This observation supports the development of age-dependent parameter assignments to characterise treatment efficacy, and corresponding rates of resistance emergence among the paediatric population.

Several published models have explored the issue of antiviral resistance induced by drug selection pressure. Within small populations, drugs may be distributed to a large proportion of the susceptible pool at once to good effect [4] [10], but such an intervention is not realistically achievable on a larger scale [6]. While Debarre's metapopulation model extended on earlier work to consider the impact of large-scale population structure on epidemic dynamics, prophylaxis was still uniformly distributed at a fixed point in time [8]. Moghadas derived optimal treatment levels for minimising the impact of drug resistance by considering the mutation process in more detail, but did not account for prophylaxis [42]. Ferguson introduced the notion of a ‘contact’ pool in order to target antiviral prophylaxis, but assumed only two contacts per infectious individual, both drawn from the susceptible class [5]. As we have previously demonstrated, underestimating the number of contacts in this manner may over-estimate the effectiveness of the intervention [1]. Lipsitch allowed for more targeted effects of interventions by considering that a fraction of exposed susceptible hosts had received prophylaxis, with variable efficacy against infection and clinical disease [7]. In contrast to our model, however, no constraint was placed on the number of doses available, resulting in drug availability throughout the entire course of the epidemic. Among other consequences, this lack of constraint facilitated the eventual emergence of resistant strain outbreaks [7].

Where asymptomatic infections make a large contribution to disease transmission, as in Ferguson's seasonal influenza model, a relatively small proportion of all infected cases and their contacts receive antivirals, making emergence of resistance less likely [5]. Conversely, when R_0_ is reduced, either by increasing the symptomatic proportion or through social distancing measures [7] including population fragmentation [8], the proportion of resistant virus strains increases due to the prolonged duration of the epidemic. Further, in larger populations where there is more time for propagation of resistant strains to occur, prophylaxis consistently favours emergence of mutant strains, with dynamic consequences contingent on fitness. Mutants with fitness above a definable but steep threshold are likely to predominate early under drug selective pressure [7]. When resistant viruses are poorly transmissible, Lipsitch concurs with our finding of extended delays to outbreak [7]. Within the time constraints of an outbreak occurring within a small susceptible pool, treatment may be more potent at inducing resistance than prophylaxis [4] [10], depending on the relative fitness and seeding rate assigned to emergent drug-resistant viruses [6].

Combined prophylaxis and treatment strategies offer improved prospects for containment of epidemic growth using antiviral agents in the event of an influenza outbreak. While resistance emergence is more likely within such a strategy, the implications for epidemic control are strongly dependent on the relative fitness of mutant strains, with the potential for either reduced or extended delays to an uncontrolled outbreak. Where two drugs are available, strategies that allocate different drugs to treated cases and their close contacts are likely to be most effective at constraining the rate of resistance emergence, thereby generally increasing the time over which epidemic growth may be contained.

We have demonstrated the critical importance of both the rate of asymptomatic infection and relative transmissibility of an emergent drug-resistant influenza virus for model predictions regarding pandemic control. Our work highlights the need for information gathering regarding these parameters, as well as more frequently described measures such as the clinical attack rate, to optimise the predictive capacity of models for use as decision support tools in the event of a pandemic.

## Supporting Information

Appendix S1 (0.72 MB PDF)
