# Supplementary material for: Impact of Emerging Antiviral Drug Resistance on Influenza Containment and Spread: Influence of Subclinical Infection and Strategic Use of a Stockpile Containing One or Two Drugs
Source: PLoS One. 2008 Jun 4;3(6):e2362. doi: 10.1371/journal.pone.0002362 (PMC2390853; doi:10.1371/journal.pone.0002362)
Supplement: Appendix S1 — (0.72 MB PDF) [file pone.0002362.s001.pdf]

Impact of emerging antiviral drug resistance on influenza containment and spread: influence of subclinical infection and strategic use of a stockpile containing one or two drugs: Appendix S1

James M. McCaw*†, James G. Wood‡, Christopher T. McCaw* and Jodie McVernon*

May 2, 2008

Contents

S1 Details of the model **2**
S1.1 The single-drug model . . . 2
S1.1.1 ODEs . . . 6
S1.1.2 R_0 calculation . . . 7
S1.2 Multi-drug models . . . 8
S1.2.1 Random allocation and cycling models . . . 8
S1.2.2 Treatment and Prophylaxis model . . . 15

S2 Results **19**
S2.1 Alternative scenarios . . . 19
S2.1.1 Extremely high fitness and lower seeding . . . 19
S2.1.2 A reduced symptomatic proportion . . . 21
S2.2 Sensitivity Analysis . . . 21
S2.2.1 Single drug model . . . 22
S2.2.2 Random allocation model . . . 24
S2.2.3 Treatment and Prophylaxis model . . . 26
S2.2.4 Comparing models . . . 27

A Multi-drug model construction **28**
A.1 Random allocation model . . . 28
A.2 Treatment and Prophylaxis model . . . 39

References **44**

*Vaccine and Immunisation Research Group, Murdoch Childrens Research Institute and Melbourne School of Population Health, The University of Melbourne, Victoria, Australia.

†Corresponding author. Electronic mail: jamesm@unimelb.edu.au, Phone: +61 3 8344 9145, Fax: +61 3 9348 1827.

‡School of Public Health & Community Medicine, University of New South Wales and National Centre for Immunisation Research and Surveillance of Vaccine Preventable Diseases, The Childrens Hospital at Westmead and the University of Sydney, New South Wales, Australia.

Abstract

We provide details of the models used in the main text, explain some of the results in greater detail, perform a sensitivity analysis for each model and explore a number of alternate scenarios to demonstrate the general nature of the results presented in the main text.

S1 Details of the model

The models used in this paper are extensions to the contact model described in detail in McCaw and McVernon (2007). Here we detail how asymptomatic infections and development of antiviral resistance is accounted for within the single-drug model. We also provide R_0 calculations for the single-drug model. The multi-drug model equations are presented for reference, while details of their construction are left to Appendix A.

S1.1 The single-drug model

We have states I_p^y with $y \in \{w, r\}$ representing symptomatic infections for those taking prophylaxis (p) and infected with the wild-type ($y = w$) or resistant-type ($y = r$) strain. States A_p^y represent equivalent asymptomatic infections.

We also have the state $I_{np,t}^w$ representing symptomatic infections with the wild-type strain for those *not* taking prophylaxis (np), but being provided with antiviral drugs as treatment (t). For symptomatic infections with the wild-type strain that are not provided with antiviral drugs at all, we have the state $I_{np,nt}^w$. Symptomatic infections with the resistant-type strain may or may not be provided with treatment but it has no effect so we require just one state I_{np}^r. Asymptomatic infections are not able to be identified and provided with antiviral drugs and so we only require two states A_{np}^y with $y \in \{w, r\}$.

The force of infection arises from each of these nine infectious classes:

$$\lambda_p^w = \beta e_i \left(I_p^w + \chi A_p^w\right) \tag{S1}$$

$$\lambda_{np,nt}^w = \beta \left(I_{np,nt}^w + \chi A_{np}^w\right) \tag{S2}$$

$$\lambda_{np,t}^w = \beta e_t I_{np,t}^w \tag{S3}$$

$$\lambda^r = \beta \phi \left(I_p^r + I_{np}^r + \chi \left(A_p^r + A_{np}^r\right)\right), \tag{S4}$$

where ϕ is the relative transmissibility of the resistant strain relative to uncontrolled wild-type and χ is the relative transmissibility of asymptomatic infections. Other parameters are defined as in McCaw and McVernon (2007).

Infections arising from these nine states cause susceptible individuals (S) to become exposed (E_x^y with $x \in \{p, np\}$ and $y \in \{w, r\}$). Exposed individuals will become infectious after a latent period of mean duration $1/\omega$ days. New infections (I and A states) have contacts (C_x^y states) which may or may not be exposed. The mean infectious period is $1/\gamma$ days.

A susceptible, upon exposure, can proceed down a number of paths, dependent upon the characteristics of the infectee (wild-type, resistant-type) and whether or not they are provided with post-exposure prophylaxis. A proportion of exposures, α, are symptomatic. Our model allows for this proportion to depend upon the nature of the strain (w or r) and the provision of prophylaxis (p or np).

Resistance development ("seeding") is captured by two parameters. A proportion, ρ_t, of those exposed to wild-type virus and treated ($\alpha_{np}^w \psi E_{np}^w$) convert to become symptomatic resistant-strain

infectors (I^r_{np}). A proportion, ρ_p, of those exposed to wild-type virus and taking prophylaxis (E^w_p) convert to become either symptomatic or asymptomatic resistant-strain infectors (I^r_p or A^r_p).

Figures S1 and S2 show the possible paths a susceptible can take on becoming infectious. These diagrams are used to construct the model equations that follow.

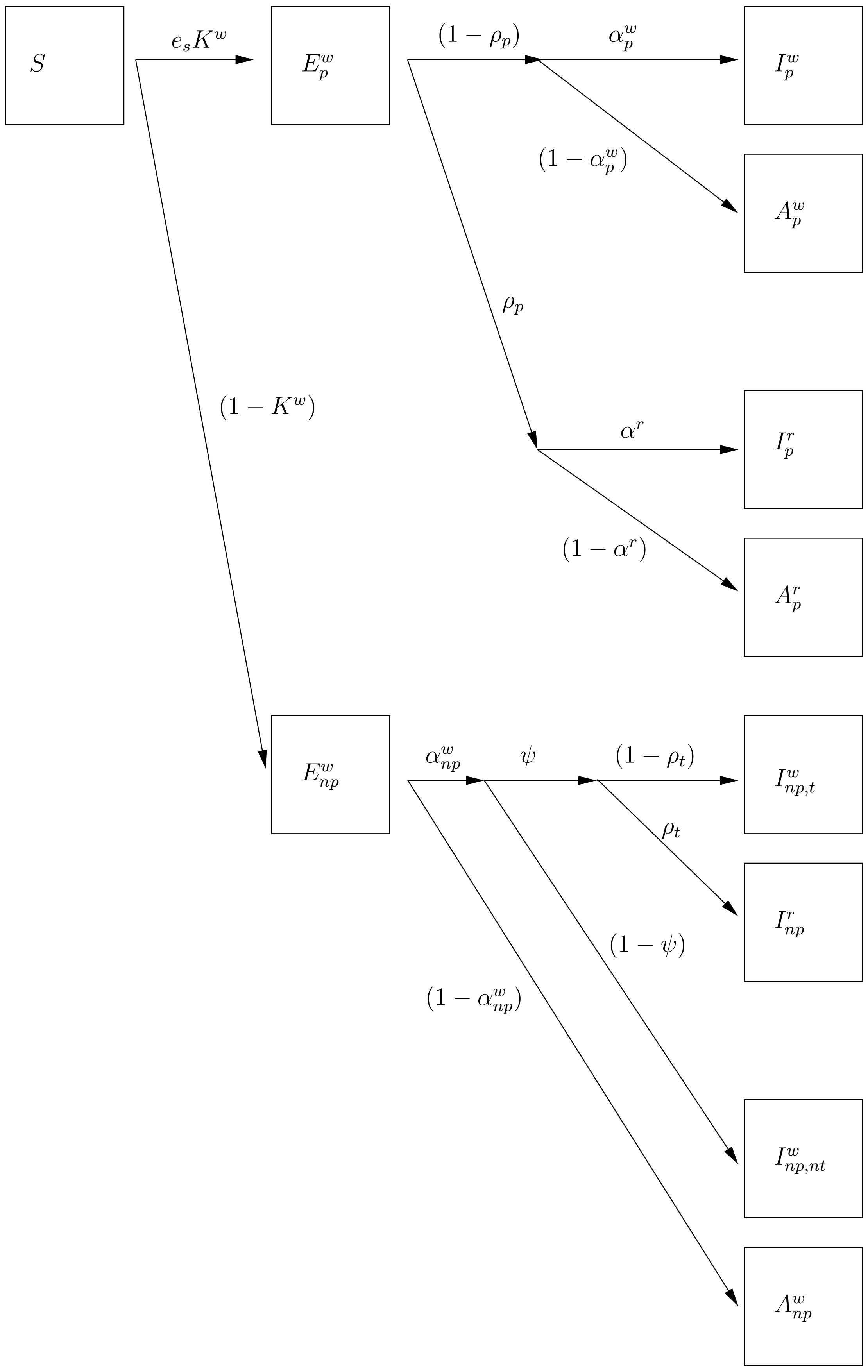

Figure S1: Tree diagram for wild strain exposures

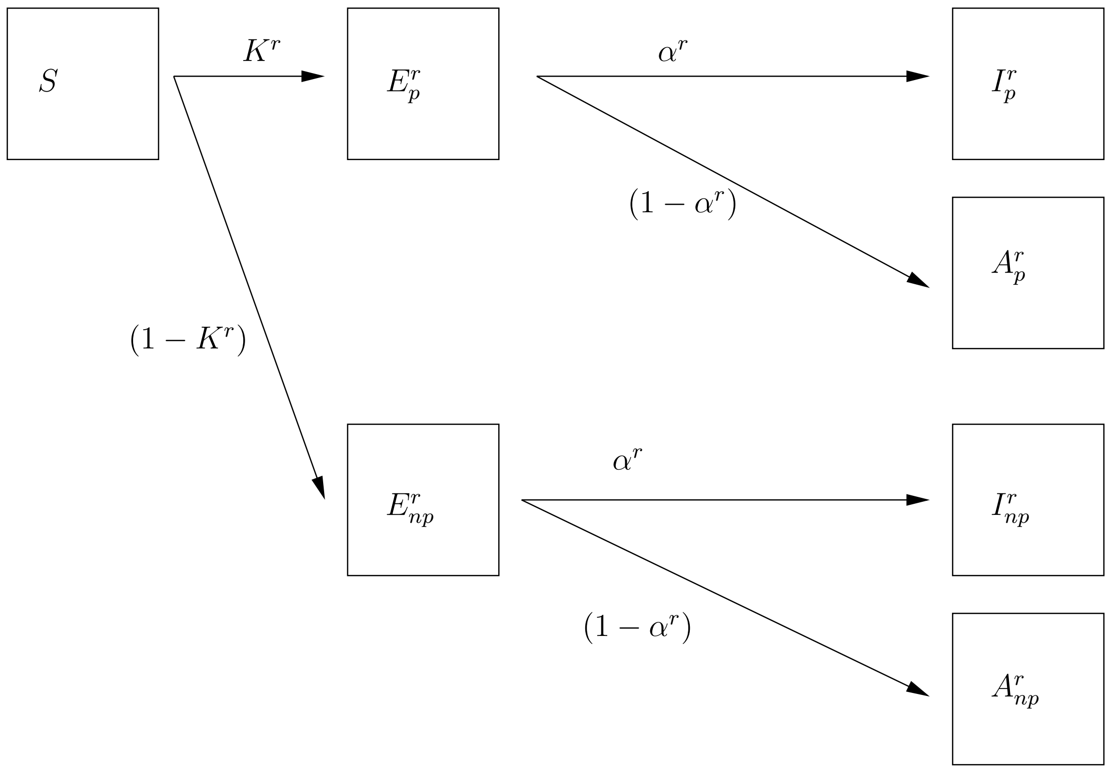

Figure S2: Tree diagram for resistant strain exposures

We introduce four contact states C_x^y ($x \in \{p, np\}$, $y \in \{w, r\}$) which classify a contact by their own type if they were to become infectious. We introduce:

$$\Theta_p^w = \frac{e_s C_p^w}{C_p^w + C_{np}^w} \frac{S}{N} \tag{S5}$$

$$\Theta_{np}^w = \frac{C_{np}^w}{C_p^w + C_{np}^w} \frac{S}{N} \tag{S6}$$

$$\Theta_p^r = \frac{C_p^r}{C_p^r + C_{np}^r} \frac{S}{N} \tag{S7}$$

$$\Theta_{np}^r = \frac{C_{np}^r}{C_p^r + C_{np}^r} \frac{S}{N}. \tag{S8}$$

Contacts of wild-type infectives who are provided with AV drugs (C_p^w) have a reduced susceptibility accounted for by the factor e_s in the equation for Θ_p^w. All other contacts (those not on prophylaxis and those on prophylaxis but in contact with resistant-strain infectives) remain fully susceptible to infection.

S1.1.1 ODEs

The model equations are:

$$\frac{dS}{dt} = -\left[\lambda^w\left(\Theta_p^w + \Theta_{np}^w\right) + \lambda^r\left(\Theta_p^r + \Theta_{np}^r\right)\right] \tag{S9}$$

$$\frac{dC_p^w}{dt} = \kappa\epsilon\omega\left\{\alpha_p^w\left(1-\rho_p\right)E_p^w + \left[\left(1-\rho_t\right)\psi + \left(1-\psi\right)\right]\alpha_{np}^w E_{np}^w\right\} - \delta C_p^w - \lambda^w\Theta_p^w \tag{S10}$$

$$\begin{aligned}\frac{dC_{np}^w}{dt} &= \kappa\left(1-\epsilon\right)\omega\left\{\alpha_p^w\left(1-\rho_p\right)E_p^w + \left[\left(1-\rho_t\right)\psi + \left(1-\psi\right)\right]\alpha_{np}^w E_{np}^w\right\} \\ &\quad + \kappa\omega\left\{\left(1-\alpha_p^w\right)\left(1-\rho_p\right)E_p^w + \left(1-\alpha_{np}^w\right)E_{np}^w\right\} - \delta C_{np}^w - \lambda^w\Theta_{np}^w\end{aligned} \tag{S11}$$

$$\frac{dC_p^r}{dt} = \kappa\epsilon\omega\left\{\alpha^r\left(E_p^r + E_{np}^r\right) + \alpha^r\rho_p E_p^w + \alpha_{np}^w\rho_t\psi E_{np}^w\right\} - \delta C_p^r - \lambda^r\Theta_p^r \tag{S12}$$

$$\begin{aligned}\frac{dC_{np}^r}{dt} &= \kappa\left(1-\epsilon\right)\omega\left\{\alpha^r\left(E_p^r + E_{np}^r\right) + \alpha^r\rho_p E_p^w + \alpha_{np}^w\rho_t\psi E_{np}^w\right\} \\ &\quad + \kappa\omega\left\{\left(1-\alpha^r\right)\left(E_p^r + E_{np}^r\right) + \left(1-\alpha^r\right)\rho_p E_p^w\right\} - \delta C_{np}^r - \lambda^r\Theta_{np}^r\end{aligned} \tag{S13}$$

and, for $x \in \{p, np\}$, $y \in \{w, r\}$

$$\frac{dE_x^y}{dt} = \lambda^y\Theta_x^y - \omega E_x^y \tag{S14}$$

and, for symptomatic infectious states

$$\frac{dI_p^w}{dt} = \alpha_p^w\left(1-\rho_p\right)\omega E_p^w - \gamma_I I_p^w \tag{S15}$$

$$\frac{dI_{np,t}^w}{dt} = \alpha_{np}^w\left(1-\rho_t\right)\psi\omega E_{np}^w - \gamma_I I_{np,t}^w \tag{S16}$$

$$\frac{dI_{np,nt}^w}{dt} = \alpha_{np}^w\left(1-\psi\right)\omega E_{np}^w - \gamma_I I_{np,nt}^w \tag{S17}$$

$$\frac{dI_p^r}{dt} = \alpha^r\rho_p\omega E_p^w + \alpha^r\omega E_p^r - \gamma_I I_p^r \tag{S18}$$

$$\frac{dI_{np}^r}{dt} = \alpha_{np}^w\rho_t\psi\omega E_{np}^w + \alpha^r\omega E_{np}^r - \gamma_I I_{np}^r \tag{S19}$$

and, for asymptomatic infectious states

$$\frac{dA_p^w}{dt} = \left(1-\alpha_p^w\right)\left(1-\rho_p\right)\omega E_p^w - \gamma_A A_p^w \tag{S20}$$

$$\frac{dA_{np}^w}{dt} = \left(1-\alpha_{np}^w\right)\omega E_{np}^w - \gamma_A A_{np}^w \tag{S21}$$

$$\frac{dA_p^r}{dt} = \left(1-\alpha^r\right)\rho_p\omega E_p^w + \left(1-\alpha^r\right)\omega E_p^r - \gamma_A A_p^r \tag{S22}$$

$$\frac{dA_{np}^r}{dt} = \left(1-\alpha^r\right)\omega E_{np}^r - \gamma_A A_{np}^r \tag{S23}$$

and, for $x \in \{p, np\}$, $y \in \{w, r\}$

$$\frac{dR_{Ix}^y}{dt} = \gamma_I I_x^y \tag{S24}$$

$$\frac{dR_{Ax}^y}{dt} = \gamma_A A_x^y \tag{S25}$$

and finally, for the depletion of the finite stockpile of AV drugs,

$$\frac{dO}{dt} = -\kappa\epsilon\omega\left[\alpha_p^w E_p^w + \alpha_{np}^w E_{np}^w + \alpha^r\left(E_p^r + E_{np}^r\right)\right] - \psi\omega\left[\alpha_{np}^w E_{np}^w + \alpha^r E_{np}^r\right]. \tag{S26}$$

S1.1.2 R_0 calculation

Define the column vector ($'$ is transpose)

$$\vec{X} = \begin{bmatrix} I_p^w & I_{np,t}^w & I_{np,nt}^w & I_p^r & I_{np}^r & A_p^w & A_{np}^w & A_p^r & A_{np}^r \end{bmatrix}' \tag{S27}$$

We may ignore the E states by factoring them into the right hand side. Defining this construction ($\dot{\vec{Y}} \equiv \dot{\vec{X}} +$ appropriate parts of $\dot{\vec{E}}$) allows us to write:

$$\dot{\vec{Y}} = \left\{[9\times 9]/\gamma - \vec{I}\right\}\gamma\vec{X} \tag{S28}$$

and R_0 is the maximum eigenvalue of the 9×9 matrix. We construct this matrix as a linear combination of tensor products of force of infection row vectors, $\vec{\lambda^w}$ and $\vec{\lambda^r}$, with suitable column vectors constructed from the differential equations, $\vec{F^w}$ and $\vec{F^r}$:

$$R_0 = \text{max eigenvalue of}\left\{\left(\vec{F^w}\vec{\lambda^w} + \vec{F^r}\vec{\lambda^r}\right)/\gamma\right\}, \tag{S29}$$

where

$$\vec{\lambda^w} = \beta\begin{bmatrix} e_i & e_t & 1 & 0 & 0 & e_i\chi & \chi & 0 & 0 \end{bmatrix} \tag{S30}$$

$$\vec{\lambda^r} = \beta\phi\begin{bmatrix} 0 & 0 & 0 & 1 & 1 & 0 & 0 & \chi & \chi \end{bmatrix}, \tag{S31}$$

and

$$\vec{F^w} = \begin{bmatrix} \alpha_p^w(1-\rho_p)e_sK^w \\ \alpha_{np}^w(1-\rho_t)\psi(1-K^w) \\ \alpha_{np}^w(1-\psi)(1-K^w) \\ \alpha^r\rho_pe_sK^w \\ \alpha_{np}^w\rho_t\psi(1-K^w) \\ \left(1-\alpha_p^w\right)(1-\rho_p)e_sK^w \\ \left(1-\alpha_{np}^w\right)(1-K^w) \\ (1-\alpha^r)\rho_pe_sK^w \\ 0 \end{bmatrix} \text{ and } \vec{F^r} = \begin{bmatrix} 0 \\ 0 \\ 0 \\ \alpha^rK^r \\ \alpha^r(1-K^r) \\ 0 \\ 0 \\ (1-\alpha^r)K^r \\ (1-\alpha^r)(1-K^r) \end{bmatrix}, \tag{S32}$$

where K^w and K^r are the steady state proportions of $C_p^x/\left(C_p^x + C_{np}^x\right)$ ($x \in \{w, r\}$), which, in the case $\alpha_p^w = \alpha_{np}^w$, are simply $\alpha_{np}^x\epsilon$ ($x \in \{w, r\}$).

The matrix $\vec{F^w}\vec{\lambda^w} + \vec{F^r}\vec{\lambda^r}$ can be put into block-triangular form, effectively breaking the eignevalue calculation into two separate calculations, one from each of $\vec{F^w}\vec{\lambda^w}$ and $\vec{F^r}\vec{\lambda^r}$. As each of these matrices is, by construction, rank-1 the eigenvalues of the system are given by the scalar product of $\vec{F^x}$ and $\vec{\lambda^x}$ ($x \in \{w, r\}$). From $x = w$, we obtain the reproduction number for the controlled wild strain; from $x = r$, the reproduction number for the reduced-fitness resistant strain:

$$R_0 = \frac{\beta}{\gamma}\times\max\left\{\begin{array}{l} \left(\alpha_p^w + \left(1-\alpha_p^w\right)\chi\right)(1-\rho_p)e_se_iK^w \\ \qquad + \left\{\alpha_{np}^w\left[e_t(1-\rho_t)\psi + (1-\psi)\right] + \left(1-\alpha_{np}^w\right)\chi\right\}(1-K^w), \\ \phi\left(\alpha^r + (1-\alpha^r)\chi\right) \end{array}\right. \tag{S33}$$

This expression deserves some explanation. The first eigenvalue is for the wild-type strain. It's form is the appropriate linear combination of prophylaxis (K^w) and non-prophylaxis ($1 - K^w$) sectors. The prophylaxis sector is itself a linear combination of symptomatic and asymptomatic parts, capturing the reduced susceptibility (e_s) and reduced infectivity (e_i) due to prophylaxis, as well as the leakage to the resistant sector. The non-prophylaxis sector is, again, a linear combination of symptomatic and asymptomatic parts. Furthermore, the symptomatic part is itself a linear combination of treated (ψ) and untreated ($1 - \psi$) components. The treatment part captures leakage to the resistant sector.

The second reproduction number is for the resistant strain. It is the appropriate linear combination of reproduction numbers for the symptomatic resistant-strain infections ($\beta\phi/\gamma$) and asymptomatic resistant-strain infections ($\beta\phi\chi/\gamma$). The simple form arises due to the fact that antiviral drugs have no impact on the resistant strain.

S1.2 Multi-drug models

The multi-drug models build upon the single-drug model just detailed. More states are included to account for the four possible strains in cicurlation (wild-type, two single-drug resistant types and multi-drug resistant type). Parameters relating to the resistant strain (ϕ, ρ_t and ρ_p as well as α^r) pick up labels to tie them to the drug in use and the strain in circulation.

S1.2.1 Random allocation and cycling models

We have four strains in circulation: with wild-type virus (w label), drug 1 resistant virus (r_1 label), drug 2 resistant virus (r_2 label) and multiple resistant virus (r_{12} label). We introduce new parameters:

- $\phi^{r_1,r_2,r_{12}}$ — the transmissibility of the (drug 1, drug 2, multiple)-resistant strain relative to the transmissibility of the uncontrolled wild-type strain.

- $\rho_{t1,t2}$ — the proportion of offspring (that is, secondary infectives) of treated wild-type infectives who carry the drug 1 (drug 2) resistant strain.

- $\rho_{p1,p2}$ — the proportion of offspring of breakthrough (prophylaxis) wild-type infectives who carry the drug 1 (drug 2) resistant strain.

We assume that all offspring of r_1-resistant infectives are either r_1-resistant or r_{12}-resistant. Similarly, all offspring of r_2-resistant infectives are either r_2-resistant or r_{12}-resistant. All offspring of r_{12}-resistant infectives are r_{12}-resistant.

The provision of AV drug 1 (drug 2) as prophylaxis to contacts of drug 2 (drug 1) resistant infectives is assumed to have the same protective effect as provision of prophylaxis to contacts of wild-type infectives. Prophylaxis with drug 1 (drug 2) of contacts of drug 1 (drug 2) resistant infectives has no protective effect. Similarly, the provision of AV drug 1 (drug 2) as treatment to drug 2 (drug 1) resistant infectives is assumed to have the same effect as provision of treatment to wild-type infectives. Treatment with drug 1 (drug 2) has no effect on infectives carrying the drug 1 (drug 2) resistant strain. Provision of AV drugs as prophylaxis to contacts or as treatment to infectives carrying the multiple-resistant strain is assumed to have no effect on either infectiousness or susceptibility.

In the random allocation model, which drug is used for treatment or prophylaxis is chosen at random, assumed to be a function of the stockpile size of each drug. For treatment or prophylaxis,

drug 1 will be provided a proportion $f_1(O_1, O_2)$ of the time and drug 2 a proportion $f_2(O_1, O_2)$ of the time. In the simplest case, f_1 and f_2 are given by the proportion of the stockpile that is of type 1 or 2:

$$f_1 = O_1/(O_1 + O_2) \tag{S34}$$

$$f_2 = O_2/(O_1 + O_2). \tag{S35}$$

In the cycling model, when using drug 1 we set $f_1 = 1$, $f_2 = 0$ and vice-versa when using drug 2.

The force of infection arises from 30 infectors:

$$\lambda_{p1}^{w} = \beta e_{i1}\left(I_{p1}^{w} + \chi A_{p1}^{w}\right) \tag{S36}$$

$$\lambda_{p2}^{w} = \beta e_{i2}\left(I_{p2}^{w} + \chi A_{p2}^{w}\right) \tag{S37}$$

$$\lambda_{np,t1}^{w} = \beta e_{t1} I_{np,t1}^{w} \tag{S38}$$

$$\lambda_{np,t2}^{w} = \beta e_{t2} I_{np,t2}^{w} \tag{S39}$$

$$\lambda_{np,nt}^{w} = \beta\left(I_{np,nt}^{w} + \chi A_{np}^{w}\right) \tag{S40}$$

$$\lambda_{p1}^{r_1} = \beta \phi^{r_1}\left(I_{p1}^{r_1} + \chi A_{p1}^{r_1}\right) \tag{S41}$$

$$\lambda_{p2}^{r_1} = \beta \phi^{r_1} e_{i2}\left(I_{p2}^{r_1} + \chi A_{p2}^{r_1}\right) \tag{S42}$$

$$\lambda_{np,t1}^{r_1} = \beta \phi^{r_1} I_{np,t1}^{r_1} \tag{S43}$$

$$\lambda_{np,t2}^{r_1} = \beta \phi^{r_1} e_{t2} I_{np,t2}^{r_1} \tag{S44}$$

$$\lambda_{np,nt}^{r_1} = \beta \phi^{r_1}\left(I_{np,nt}^{r_1} + \chi A_{np}^{r_1}\right) \tag{S45}$$

$$\lambda_{p1}^{r_2} = \beta \phi^{r_2} e_{i1}\left(I_{p1}^{r_2} + \chi A_{p1}^{r_2}\right) \tag{S46}$$

$$\lambda_{p2}^{r_2} = \beta \phi^{r_2}\left(I_{p2}^{r_2} + \chi A_{p2}^{r_2}\right) \tag{S47}$$

$$\lambda_{np,t1}^{r_2} = \beta \phi^{r_2} e_{t1} I_{np,t1}^{r_2} \tag{S48}$$

$$\lambda_{np,t2}^{r_2} = \beta \phi^{r_2} I_{np,t2}^{r_2} \tag{S49}$$

$$\lambda_{np,nt}^{r_2} = \beta \phi^{r_2}\left(I_{np,nt}^{r_2} + \chi A_{np}^{r_2}\right) \tag{S50}$$

$$\lambda^{r_{12}} = \beta \phi^{r_{12}}\left(I_{p1}^{r_{12}} + I_{p2}^{r_{12}} + I_{np}^{r_{12}} + \chi\left(A_{p1}^{r_{12}} + A_{p2}^{r_{12}} + A_{np}^{r_{12}}\right)\right). \tag{S51}$$

Compared to the single-drug model, we now have twelve contact states C_x^y ($x \in \{p1, p2, np\}$, $y \in \{w, r_1, r_2, r_{12}\}$):

$$\Theta_{p1}^{w} = \frac{e_{s1} C_{p1}^{w}}{C_{p1}^{w} + C_{p2}^{w} + C_{np}^{w}} \frac{S}{N} \tag{S52}$$

$$\Theta_{p2}^{w} = \frac{e_{s2} C_{p2}^{w}}{C_{p1}^{w} + C_{p2}^{w} + C_{np}^{w}} \frac{S}{N} \tag{S53}$$

$$\Theta_{np}^{w} = \frac{C_{np}^{w}}{C_{p1}^{w} + C_{p2}^{w} + C_{np}^{w}} \frac{S}{N} \tag{S54}$$

$$\Theta_{p1}^{r_1} = \frac{C_{p1}^{r_1}}{C_{p1}^{r_1} + C_{p2}^{r_1} + C_{np}^{r_1}} \frac{S}{N} \tag{S55}$$

$$\Theta_{p2}^{r_1} = \frac{e_{s2} C_{p2}^{r_1}}{C_{p1}^{r_1} + C_{p2}^{r_1} + C_{np}^{r_1}} \frac{S}{N} \tag{S56}$$

$$\Theta_{np}^{r_1} = \frac{C_{np}^{r_1}}{C_{p1}^{r_1} + C_{p2}^{r_1} + C_{np}^{r_1}} \frac{S}{N} \tag{S57}$$

$$\Theta_{p1}^{r_2} = \frac{e_{s1} C_{p1}^{r_2}}{C_{p1}^{r_2} + C_{p2}^{r_2} + C_{np}^{r_2}} \frac{S}{N} \tag{S58}$$

$$\Theta_{p2}^{r_2} = \frac{C_{p2}^{r_2}}{C_{p1}^{r_2} + C_{p2}^{r_2} + C_{np}^{r_2}} \frac{S}{N} \tag{S59}$$

$$\Theta_{np}^{r_2} = \frac{C_{np}^{r_2}}{C_{p1}^{r_2} + C_{p2}^{r_2} + C_{np}^{r_2}} \frac{S}{N} \tag{S60}$$

$$\Theta_{p1}^{r_{12}} = \frac{C_{p1}^{r_{12}}}{C_{p1}^{r_{12}} + C_{p2}^{r_{12}} + C_{np}^{r_{12}}} \frac{S}{N} \tag{S61}$$

$$\Theta_{p2}^{r_{12}} = \frac{C_{p2}^{r_{12}}}{C_{p1}^{r_{12}} + C_{p2}^{r_{12}} + C_{np}^{r_{12}}} \frac{S}{N} \tag{S62}$$

$$\Theta_{np}^{r_{12}} = \frac{C_{np}^{r_{12}}}{C_{p1}^{r_{12}} + C_{p2}^{r_{12}} + C_{np}^{r_{12}}} \frac{S}{N} \tag{S63}$$

.

The differential equations that describe the dynamics are:

$$\frac{dS}{dt} = -\left[\lambda^{w}\left(\Theta_{p1}^{w} + \Theta_{p2}^{w} + \Theta_{np}^{w}\right) + \lambda^{r_1}\left(\Theta_{p1}^{r_1} + \Theta_{p2}^{r_1} + \Theta_{np}^{r_1}\right) + \lambda^{r_2}\left(\Theta_{p1}^{r_2} + \Theta_{p2}^{r_2} + \Theta_{np}^{r_2}\right) + \lambda^{r_{12}}\left(\Theta_{p1}^{r_{12}} + \Theta_{p2}^{r_{12}} + \Theta_{np}^{r_{12}}\right)\right] \tag{S64}$$

and for the wild-type contact classes

$$\frac{dC_{p1}^{w}}{dt} = \kappa\epsilon\omega f_1 \left\{\alpha_{p1}^{w}\left(1-\rho_{p1}\right)E_{p1}^{w} + \alpha_{p2}^{w}\left(1-\rho_{p2}\right)E_{p2}^{w} \right. \\ \left. + \left[\left(1-\rho_{t1}\right)f_1\psi + \left(1-\rho_{t2}\right)f_2\psi + \left(1-\psi\right)\right]\alpha_{np}^{w}E_{np}^{w}\right\} - \delta C_{p1}^{w} - \lambda^{w}\Theta_{p1}^{w} \quad \text{(S65)}$$

$$\frac{dC_{p2}^{w}}{dt} = \kappa\epsilon\omega f_2 \left\{\alpha_{p1}^{w}\left(1-\rho_{p1}\right)E_{p1}^{w} + \alpha_{p2}^{w}\left(1-\rho_{p2}\right)E_{p2}^{w} \right. \\ \left. + \left[\left(1-\rho_{t1}\right)f_1\psi + \left(1-\rho_{t2}\right)f_2\psi + \left(1-\psi\right)\right]\alpha_{np}^{w}E_{np}^{w}\right\} - \delta C_{p2}^{w} - \lambda^{w}\Theta_{p2}^{w} \quad \text{(S66)}$$

$$\frac{dC_{np}^{w}}{dt} = \kappa\left(1-\epsilon\right)\omega \left\{\alpha_{p1}^{w}\left(1-\rho_{p1}\right)E_{p1}^{w} + \alpha_{p2}^{w}\left(1-\rho_{p2}\right)E_{p2}^{w} \right. \\ \left. + \left[\left(1-\rho_{t1}\right)f_1\psi + \left(1-\rho_{t2}\right)f_2\psi + \left(1-\psi\right)\right]\alpha_{np}^{w}E_{np}^{w}\right\} \\ + \kappa\omega\left\{\left(1-\alpha_{p1}^{w}\right)\left(1-\rho_{p1}\right)E_{p1}^{w} + \left(1-\alpha_{p2}^{w}\right)\left(1-\rho_{p2}\right)E_{p2}^{w} + \left(1-\alpha_{np}^{w}\right)E_{np}^{w}\right\} \\ - \delta C_{np}^{w} - \lambda^{w}\Theta_{np}^{w} \quad \text{(S67)}$$

and for the drug 1 resistant contact classes

$$\frac{dC_{p1}^{r_1}}{dt} = \kappa\epsilon\omega f_1 \left\{\alpha_{p1}^{r_1}E_{p1}^{r_1} + \alpha_{p2}^{r_1}\left(1-\rho_{p2}\right)E_{p2}^{r_1} + \left[f_1\psi + \left(1-\rho_{t2}\right)f_2\psi + \left(1-\psi\right)\right]\alpha_{np}^{r_1}E_{np}^{r_1} \right. \\ \left. + \alpha_{p1}^{r_1}\rho_{p1}E_{p1}^{w} + \alpha_{np}^{w}\rho_{t1}f_1\psi E_{np}^{w}\right\} - \delta C_{p1}^{r_1} - \lambda^{r_1}\Theta_{p1}^{r_1} \quad \text{(S68)}$$

$$\frac{dC_{p2}^{r_1}}{dt} = \kappa\epsilon\omega f_2 \left\{\alpha_{p1}^{r_1}E_{p1}^{r_1} + \alpha_{p2}^{r_1}\left(1-\rho_{p2}\right)E_{p2}^{r_1} + \left[f_1\psi + \left(1-\rho_{t2}\right)f_2\psi + \left(1-\psi\right)\right]\alpha_{np}^{r_1}E_{np}^{r_1} \right. \\ \left. + \alpha_{p1}^{r_1}\rho_{p1}E_{p1}^{w} + \alpha_{np}^{w}\rho_{t1}f_1\psi E_{np}^{w}\right\} - \delta C_{p2}^{r_1} - \lambda^{r_1}\Theta_{p2}^{r_1} \quad \text{(S69)}$$

$$\frac{dC_{np}^{r_1}}{dt} = \kappa\left(1-\epsilon\right)\omega \left\{\alpha_{p1}^{r_1}E_{p1}^{r_1} + \alpha_{p2}^{r_1}\left(1-\rho_{p2}\right)E_{p2}^{r_1} + \left[f_1\psi + \left(1-\rho_{t2}\right)f_2\psi + \left(1-\psi\right)\right]\alpha_{np}^{r_1}E_{np}^{r_1} \right. \\ \left. + \alpha_{p1}^{r_1}\rho_{p1}E_{p1}^{w} + \alpha_{np}^{w}\rho_{t1}f_1\psi E_{np}^{w}\right\} \\ + \kappa\omega\left\{\left(1-\alpha_{p1}^{r_1}\right)E_{p1}^{r_1} + \left(1-\alpha_{p2}^{r_1}\right)\left(1-\rho_{p2}\right)E_{p2}^{r_1} + \left(1-\alpha_{np}^{r_1}\right)E_{np}^{r_1} \right. \\ \left. + \left(1-\alpha_{p1}^{r_1}\right)\rho_{p1}E_{p1}^{w}\right\} - \delta C_{np}^{r_1} - \lambda^{r_1}\Theta_{np}^{r_1} \quad \text{(S70)}$$

and for the drug 2 resistant contact classes

$$\frac{dC_{p1}^{r_2}}{dt} = \kappa\epsilon\omega f_1 \left\{\alpha_{p2}^{r_2}E_{p2}^{r_2} + \alpha_{p1}^{r_2}\left(1-\rho_{p1}\right)E_{p1}^{r_2} + \left[\left(1-\rho_{t1}\right)f_1\psi + f_2\psi + \left(1-\psi\right)\right]\alpha_{np}^{r_2}E_{np}^{r_2} \right. \\ \left. + \alpha_{p2}^{r_2}\rho_{p2}E_{p2}^{w} + \alpha_{np}^{w}\rho_{t2}f_2\psi E_{np}^{w}\right\} - \delta C_{p1}^{r_2} - \lambda^{r_2}\Theta_{p1}^{r_2} \quad \text{(S71)}$$

$$\frac{dC_{p2}^{r_2}}{dt} = \kappa\epsilon\omega f_2 \left\{\alpha_{p2}^{r_2}E_{p2}^{r_2} + \alpha_{p1}^{r_2}\left(1-\rho_{p1}\right)E_{p1}^{r_2} + \left[\left(1-\rho_{t1}\right)f_1\psi + f_2\psi + \left(1-\psi\right)\right]\alpha_{np}^{r_2}E_{np}^{r_2} \right. \\ \left. + \alpha_{p2}^{r_2}\rho_{p2}E_{p2}^{w} + \alpha_{np}^{w}\rho_{t2}f_2\psi E_{np}^{w}\right\} - \delta C_{p2}^{r_2} - \lambda^{r_2}\Theta_{p2}^{r_2} \quad \text{(S72)}$$

$$\frac{dC_{np}^{r_2}}{dt} = \kappa\left(1-\epsilon\right)\omega \left\{\alpha_{p2}^{r_2}E_{p2}^{r_2} + \alpha_{p1}^{r_2}\left(1-\rho_{p1}\right)E_{p1}^{r_2} + \left[\left(1-\rho_{t1}\right)f_1\psi + f_2\psi + \left(1-\psi\right)\right]\alpha_{np}^{r_2}E_{np}^{r_2} \right. \\ \left. + \alpha_{p2}^{r_2}\rho_{p2}E_{p2}^{w} + \alpha_{np}^{w}\rho_{t2}f_2\psi E_{np}^{w}\right\} \\ + \kappa\omega\left\{\left(1-\alpha_{p2}^{r_2}\right)E_{p2}^{r_2} + \left(1-\alpha_{p1}^{r_2}\right)\left(1-\rho_{p1}\right)E_{p1}^{r_2} + \left(1-\alpha_{np}^{r_2}\right)E_{np}^{r_2} \right. \\ \left. + \left(1-\alpha_{p2}^{r_2}\right)\rho_{p2}E_{p2}^{w}\right\} - \delta C_{np}^{r_2} - \lambda^{r_2}\Theta_{np}^{r_2} \quad \text{(S73)}$$

and for the multi-drug resistant contact classes

$$\frac{dC_{p1}^{r_{12}}}{dt} = \kappa\epsilon\omega f_1 \left\{\alpha^{r_{12}} E_{p1}^{r_{12}} + \alpha^{r_{12}} E_{p2}^{r_{12}} + \alpha^{r_{12}} E_{np}^{r_{12}} + \alpha^{r_{12}} \rho_{p2} E_{p2}^{r_1} + \alpha^{r_{12}} \rho_{p1} E_{p1}^{r_2} \right.$$
$$\left. + \alpha_{np}^{r_1} \rho_{t2} f_2 \psi E_{np}^{r_1} + \alpha_{np}^{r_2} \rho_{t1} f_1 \psi E_{np}^{r_2} \right\} - \delta C_{p1}^{r_{12}} - \lambda^{r_{12}} \Theta_{p1}^{r_{12}} \quad \text{(S74)}$$

$$\frac{dC_{p2}^{r_{12}}}{dt} = \kappa\epsilon\omega f_2 \left\{\alpha^{r_{12}} E_{p1}^{r_{12}} + \alpha^{r_{12}} E_{p2}^{r_{12}} + \alpha^{r_{12}} E_{np}^{r_{12}} + \alpha^{r_{12}} \rho_{p2} E_{p2}^{r_1} + \alpha^{r_{12}} \rho_{p1} E_{p1}^{r_2} \right.$$
$$\left. + \alpha_{np}^{r_1} \rho_{t2} f_2 \psi E_{np}^{r_1} + \alpha_{np}^{r_2} \rho_{t1} f_1 \psi E_{np}^{r_2} \right\} - \delta C_{p2}^{r_{12}} - \lambda^{r_{12}} \Theta_{p2}^{r_{12}} \quad \text{(S75)}$$

$$\frac{dC_{np}^{r_{12}}}{dt} = \kappa\left(1-\epsilon\right)\omega \left\{\alpha^{r_{12}} E_{p1}^{r_{12}} + \alpha^{r_{12}} E_{p2}^{r_{12}} + \alpha^{r_{12}} E_{np}^{r_{12}} + \alpha^{r_{12}} \rho_{p2} E_{p2}^{r_1} + \alpha^{r_{12}} \rho_{p1} E_{p1}^{r_2} \right.$$
$$\left. + \alpha_{np}^{r_1} \rho_{t2} f_2 \psi E_{np}^{r_1} + \alpha_{np}^{r_2} \rho_{t1} f_1 \psi E_{np}^{r_2} \right\}$$
$$+ \kappa\omega \left\{\left(1-\alpha^{r_{12}}\right) E_{p1}^{r_{12}} + \left(1-\alpha^{r_{12}}\right) E_{p2}^{r_{12}} + \left(1-\alpha^{r_{12}}\right) E_{np}^{r_{12}} \right.$$
$$\left. + \left(1-\alpha^{r_{12}}\right) \rho_{p2} E_{p2}^{r_1} + \left(1-\alpha^{r_{12}}\right) \rho_{p1} E_{p1}^{r_2} \right\} - \delta C_{np}^{r_{12}} - \lambda^{r_{12}} \Theta_{np}^{r_{12}} \quad \text{(S76)}$$

and, for $x \in \{p1, p2, np\}$, $y \in \{w, r_1, r_2, r_{12}\}$,

$$\frac{dE_x^y}{dt} = \lambda^y \Theta_x^y - \omega E_x^y \quad \text{(S77)}$$

and, for symptomatic infectious states

$$\frac{dI_{p1}^{w}}{dt} = \alpha_{p1}^{w}\left(1-\rho_{p1}\right)\omega E_{p1}^{w} - \gamma_I I_{p1}^{w} \tag{S78}$$

$$\frac{dI_{p2}^{w}}{dt} = \alpha_{p2}^{w}\left(1-\rho_{p2}\right)\omega E_{p2}^{w} - \gamma_I I_{p2}^{w} \tag{S79}$$

$$\frac{dI_{np,t1}^{w}}{dt} = \alpha_{np}^{w}\left(1-\rho_{t1}\right)f_1\psi\omega E_{np}^{w} - \gamma_I I_{np,t1}^{w} \tag{S80}$$

$$\frac{dI_{np,t2}^{w}}{dt} = \alpha_{np}^{w}\left(1-\rho_{t2}\right)f_2\psi\omega E_{np}^{w} - \gamma_I I_{np,t2}^{w} \tag{S81}$$

$$\frac{dI_{np,nt}^{w}}{dt} = \alpha_{np}^{w}\left(1-\psi\right)\omega E_{np}^{w} - \gamma_I I_{np,nt}^{w} \tag{S82}$$

$$\frac{dI_{p1}^{r_1}}{dt} = \alpha_{p1}^{r_1}\rho_{p1}\omega E_{p1}^{w} + \alpha_{p1}^{r_1}\omega E_{p1}^{r_1} - \gamma_I I_{p1}^{r_1} \tag{S83}$$

$$\frac{dI_{p2}^{r_1}}{dt} = \alpha_{p2}^{r_1}\left(1-\rho_{p2}\right)\omega E_{p2}^{r_1} - \gamma_I I_{p2}^{r_1} \tag{S84}$$

$$\frac{dI_{np,t1}^{r_1}}{dt} = \alpha_{np}^{w}\rho_{t1}f_1\psi\omega E_{np}^{w} + \alpha_{np}^{r_1}f_1\psi\omega E_{np}^{r_1} - \gamma_I I_{np,t1}^{r_1} \tag{S85}$$

$$\frac{dI_{np,t2}^{r_1}}{dt} = \alpha_{np}^{r_1}\left(1-\rho_{t2}\right)f_2\psi\omega E_{np}^{r_1} - \gamma_I I_{np,t2}^{r_1} \tag{S86}$$

$$\frac{dI_{np,nt}^{r_1}}{dt} = \alpha_{np}^{r_1}\left(1-\psi\right)\omega E_{np}^{r_1} - \gamma_I I_{np,nt}^{r_1} \tag{S87}$$

$$\frac{dI_{p1}^{r_2}}{dt} = \alpha_{p1}^{r_2}\left(1-\rho_{p1}\right)\omega E_{p1}^{r_2} - \gamma_I I_{p1}^{r_2} \tag{S88}$$

$$\frac{dI_{p2}^{r_2}}{dt} = \alpha_{p2}^{r_2}\rho_{p2}\omega E_{p2}^{w} + \alpha_{p2}^{r_2}\omega E_{p2}^{r_2} - \gamma_I I_{p2}^{r_2} \tag{S89}$$

$$\frac{dI_{np,t1}^{r_2}}{dt} = \alpha_{np}^{r_2}\left(1-\rho_{t1}\right)f_1\psi\omega E_{np}^{r_2} - \gamma_I I_{np,t1}^{r_2} \tag{S90}$$

$$\frac{dI_{np,t2}^{r_2}}{dt} = \alpha_{np}^{w}\rho_{t2}f_2\psi\omega E_{np}^{w} + \alpha_{np}^{r_2}f_2\psi\omega E_{np}^{r_2} - \gamma_I I_{np,t2}^{r_2} \tag{S91}$$

$$\frac{dI_{np,nt}^{r_2}}{dt} = \alpha_{np}^{r_2}\left(1-\psi\right)\omega E_{np}^{r_2} - \gamma_I I_{np,nt}^{r_2} \tag{S92}$$

$$\frac{dI_{p1}^{r_{12}}}{dt} = \alpha^{r_{12}}\rho_{p1}\omega E_{p1}^{r_2} + \alpha^{r_{12}}\omega E_{p1}^{r_{12}} - \gamma_I I_{p1}^{r_{12}} \tag{S93}$$

$$\frac{dI_{p2}^{r_{12}}}{dt} = \alpha^{r_{12}}\rho_{p2}\omega E_{p2}^{r_1} + \alpha^{r_{12}}\omega E_{p2}^{r_{12}} - \gamma_I I_{p2}^{r_{12}} \tag{S94}$$

$$\frac{dI_{np}^{r_{12}}}{dt} = \alpha_{np}^{r_1}\rho_{t2}f_2\psi\omega E_{np}^{r_1} + \alpha_{np}^{r_2}\rho_{t1}f_1\psi\omega E_{np}^{r_2} + \alpha^{r_{12}}\omega E_{np}^{r_{12}} - \gamma_I I_{np}^{r_{12}} \tag{S95}$$

and, for asymptomatic infectious states

$$\frac{dA^w_{p1}}{dt} = \left(1-\alpha^w_{p1}\right)\left(1-\rho_{p1}\right)\omega E^w_{p1} - \gamma_A A^w_{p1} \tag{S96}$$
$$\frac{dA^w_{p2}}{dt} = \left(1-\alpha^w_{p2}\right)\left(1-\rho_{p2}\right)\omega E^w_{p2} - \gamma_A A^w_{p2} \tag{S97}$$
$$\frac{dA^w_{np}}{dt} = \left(1-\alpha^w_{np}\right)\omega E^w_{np} - \gamma_A A^w_{np} \tag{S98}$$
$$\frac{dA^{r_1}_{p1}}{dt} = \left(1-\alpha^{r_1}_{p1}\right)\rho_{p1}\omega E^w_{p1} + \left(1-\alpha^{r_1}_{p1}\right)\omega E^{r_1}_{p1} - \gamma_A A^{r_1}_{p1} \tag{S99}$$
$$\frac{dA^{r_1}_{p2}}{dt} = \left(1-\alpha^{r_1}_{p2}\right)\left(1-\rho_{p2}\right)\omega E^{r_1}_{p2} - \gamma_A A^{r_1}_{p2} \tag{S100}$$
$$\frac{dA^{r_1}_{np}}{dt} = \left(1-\alpha^{r_1}_{np}\right)\omega E^{r_1}_{np} - \gamma_A A^{r_1}_{np} \tag{S101}$$
$$\frac{dA^{r_2}_{p1}}{dt} = \left(1-\alpha^{r_2}_{p1}\right)\left(1-\rho_{p1}\right)\omega E^{r_2}_{p1} - \gamma_A A^{r_2}_{p1} \tag{S102}$$
$$\frac{dA^{r_2}_{p2}}{dt} = \left(1-\alpha^{r_2}_{p2}\right)\rho_{p2}\omega E^w_{p2} + \left(1-\alpha^{r_2}_{p2}\right)\omega E^{r_2}_{p2} - \gamma_A A^{r_2}_{p2} \tag{S103}$$
$$\frac{dA^{r_2}_{np}}{dt} = \left(1-\alpha^{r_2}_{np}\right)\omega E^{r_2}_{np} - \gamma_A A^{r_2}_{np} \tag{S104}$$
$$\frac{dA^{r_{12}}_{p1}}{dt} = \left(1-\alpha^{r_{12}}\right)\rho_{p1}\omega E^{r_2}_{p1} + \left(1-\alpha^{r_{12}}\right)\omega E^{r_{12}}_{p1} - \gamma_A A^{r_{12}}_{p1} \tag{S105}$$
$$\frac{dA^{r_{12}}_{p2}}{dt} = \left(1-\alpha^{r_{12}}\right)\rho_{p2}\omega E^{r_1}_{p2} + \left(1-\alpha^{r_{12}}\right)\omega E^{r_{12}}_{p2} - \gamma_A A^{r_{12}}_{p2} \tag{S106}$$
$$\frac{dA^{r_{12}}_{np}}{dt} = \left(1-\alpha^{r_{12}}\right)\omega E^{r_{12}}_{np} - \gamma_A A^{r_{12}}_{np} \tag{S107}$$

and, for $x \in \{p1, p2, np\}$, $y \in \{w, r_1, r_2, r_{12}\}$,

$$\frac{dR^y_{Ix}}{dt} = \gamma_I I^y_x \tag{S108}$$
$$\frac{dR^y_{Ax}}{dt} = \gamma_A A^y_x \tag{S109}$$

and finally, for the depletion of the finite stockpiles of AV drugs,

$$\begin{aligned}\frac{dO_{\text{total}}}{dt} = &-\kappa\epsilon\omega\left[\alpha^w_{p1}E^w_{p1} + \alpha^w_{p2}E^w_{p2} + \alpha^w_{np}E^w_{np} + \alpha^{r_1}_{p1}E^{r_1}_{p1} + \alpha^{r_1}_{p2}E^{r_1}_{p2} + \alpha^{r_1}_{np}E^{r_1}_{np}\right.\\ &\left.+\alpha^{r_2}_{p1}E^{r_2}_{p1} + \alpha^{r_2}_{p2}E^{r_2}_{p2} + \alpha^{r_2}_{np}E^{r_2}_{np} + \alpha^{r_{12}}\left(E^{r_{12}}_{p1} + E^{r_{12}}_{p2} + E^{r_{12}}_{np}\right)\right]\\ &- \psi\omega\left[\alpha^w_{np}E^w_{np} + \alpha^{r_1}_{np}E^{r_1}_{np} + \alpha^{r_2}_{np}E^{r_2}_{np} + \alpha^{r_{12}}E^{r_{12}}_{np}\right]\end{aligned} \tag{S110}$$

with

$$\frac{dO_1}{dt} = f_1\frac{dO_{\text{total}}}{dt} \tag{S111}$$
$$\frac{dO_2}{dt} = f_2\frac{dO_{\text{total}}}{dt} \tag{S112}$$

R_0 is the maximum eigenvalue of a 30×30 matrix. As in the single-drug model, each of the four non-zero eigenvalues corresponds to the reproduction number for one of the four strains in circulation:

$$R_0 = \frac{\beta}{\gamma} \times \max \begin{cases} \left(\alpha_{p1}^{w} + \left(1-\alpha_{p1}^{w}\right)\chi\right)\left(1-\rho_{p1}\right) e_{s1} e_{i1} K_{p1}^{w} \\ \qquad + \left(\alpha_{p2}^{w} + \left(1-\alpha_{p2}^{w}\right)\chi\right)\left(1-\rho_{p2}\right) e_{s2} e_{i2} K_{p2}^{w} \\ \qquad + \left(\alpha_{np}^{w}\left[e_{t1} f_1 \left(1-\rho_{t1}\right)\psi + e_{t2} f_2 \left(1-\rho_{t2}\right)\psi + \left(1-\psi\right)\right]\right. \\ \qquad\qquad \left. + \left(1-\alpha_{np}^{w}\right)\chi\right)\left(1-K_{p1}^{w}-K_{p2}^{w}\right), \\ \phi^{r_1}\left\{\left(\alpha_{p1}^{r_1} + \left(1-\alpha_{p1}^{r_1}\right)\chi\right) K_{p1}^{r_1}\right. \\ \qquad + \left(\alpha_{p2}^{r_1} + \left(1-\alpha_{p2}^{r_1}\right)\chi\right)\left(1-\rho_{p2}\right) e_{s2} e_{i2} K_{p2}^{r_1} \\ \qquad + \left(\alpha_{np}^{r_1}\left[f_1\psi + e_{t2} f_2 \left(1-\rho_{t2}\right)\psi + \left(1-\psi\right)\right]\right. \\ \qquad\qquad \left.\left. + \left(1-\alpha_{np}^{r_1}\right)\chi\right)\left(1-K_{p1}^{r_1}-K_{p2}^{r_1}\right)\right\}, \\ \phi^{r_2}\left\{\left(\alpha_{p1}^{r_2} + \left(1-\alpha_{p1}^{r_2}\right)\chi\right)\left(1-\rho_{p1}\right) e_{s1} e_{i1} K_{p1}^{r_2}\right. \\ \qquad + \left(\alpha_{p2}^{r_2} + \left(1-\alpha_{p2}^{r_2}\right)\chi\right) K_{p2}^{r_2} \\ \qquad + \left(\alpha_{np}^{r_2}\left[e_{t1} f_1 \left(1-\rho_{t1}\right)\psi + f_2\psi + \left(1-\psi\right)\right]\right. \\ \qquad\qquad \left.\left. + \left(1-\alpha_{np}^{r_2}\right)\chi\right)\left(1-K_{p1}^{r_2}-K_{p2}^{r_2}\right)\right\}, \\ \phi^{r_{12}}\left\{\alpha^{r_{12}} + \left(1-\alpha^{r_{12}}\right)\chi\right\} \end{cases} \tag{S113}$$

S1.2.2 Treatment and Prophylaxis model

We have four strains in circulation:wild-type virus (w label), treatment-resistant virus (r_t label), prophylaxis-resistant virus (r_p label) and multi-resistant virus (r_{tp} label). We introduce new parameters:

- $\phi^{r_t, r_p, r_{tp}}$ — the transmissibility of the (treatment, prophylaxis, multiple)-resistant strain relative to the transmissibility of the uncontrolled wild-type strain.
- ρ_t — the proportion of offspring (that is, secondary infectives) of treated wild-type infectives who carry the resistant strain.
- ρ_p — the proportion of offspring of breakthrough (prophylaxis) wild-type infectives who carry the resistant strain.

We assume that all offspring of r_t-resistant infectives are either r_t-resistant or r_{tp}-resistant. Similarly, all offspring of r_p-resistant infectives are either r_p-resistant or r_{tp}-resistant. All offspring of r_{tp}-resistant infectives are r_{tp}-resistant.

The provision of AV drugs as prophylaxis to contacts of treatment-resistant infectives is assumed to have the same protective effect as provision of prophylaxis to contacts of wild-type infectives. Treatment has no effect on infectives carrying the treatment-resistant strain. Similarly, the provision of AV drugs as treatment to prophylaxis-resistant infectives is assumed to have the same effect as provision of treatment to wild-type infectives. Prophylaxis of contacts of prophylaxis-resistant infectives has no protective effect.

Provision of AV drugs as prophylaxis to contacts or as treatment to infectives carrying the multiple-resistant strain is assumed to have no effect on either infectiousness or susceptibility.

The force of infections arises from 18 infectors:

$$\lambda_p^w = \beta e_i \left(I_p^w + \chi A_p^w\right) \tag{S114}$$
$$\lambda_{np,t}^w = \beta e_t I_{np,t}^w \tag{S115}$$
$$\lambda_{np,nt}^w = \beta \left(I_{np,nt}^w + \chi A_{np}^w\right) \tag{S116}$$
$$\lambda_p^{r_p} = \beta \phi^{r_p} \left(I_p^{r_p} + \chi A_p^{r_p}\right) \tag{S117}$$
$$\lambda_{np,t}^{r_p} = \beta \phi^{r_p} e_t I_{np,t}^{r_p} \tag{S118}$$
$$\lambda_{np,nt}^{r_p} = \beta \phi^{r_p} \left(I_{np,nt}^{r_p} + \chi A_{np}^{r_p}\right) \tag{S119}$$
$$\lambda_p^{r_t} = \beta \phi^{r_t} e_i \left(I_p^{r_t} + \chi A_p^{r_t}\right) \tag{S120}$$
$$\lambda_{np}^{r_t} = \beta \phi^{r_t} \left(I_{np}^{r_t} + \chi A_{np}^{r_t}\right) \tag{S121}$$
$$\lambda^{r_{tp}} = \beta \phi^{r_{tp}} \left(I_p^{r_{tp}} + I_{np}^{r_{tp}} + \chi \left(A_p^{r_{tp}} + A_{np}^{r_{tp}}\right)\right). \tag{S122}$$

We have eight contact states: C_x^y ($x \in \{p, np\}$, $y \in \{w, r_t, r_p, r_{tp}\}$):

$$\Theta_p^w = \frac{e_s C_p^w}{C_p^w + C_{np}^w} \frac{S}{N} \tag{S123}$$
$$\Theta_{np}^w = \frac{C_{np}^w}{C_p^w + C_{np}^w} \frac{S}{N} \tag{S124}$$
$$\Theta_p^{r_p} = \frac{C_p^{r_p}}{C_p^{r_p} + C_{np}^{r_p}} \frac{S}{N} \tag{S125}$$
$$\Theta_{np}^{r_p} = \frac{C_{np}^{r_p}}{C_p^{r_p} + C_{np}^{r_p}} \frac{S}{N} \tag{S126}$$
$$\Theta_p^{r_t} = \frac{e_s C_p^{r_t}}{C_p^{r_t} + C_{np}^{r_t}} \frac{S}{N} \tag{S127}$$
$$\Theta_{np}^{r_t} = \frac{C_{np}^{r_t}}{C_p^{r_t} + C_{np}^{r_t}} \frac{S}{N} \tag{S128}$$
$$\Theta_p^{r_{tp}} = \frac{C_p^{r_{tp}}}{C_p^{r_{tp}} + C_{np}^{r_{tp}}} \frac{S}{N} \tag{S129}$$
$$\Theta_{np}^{r_{tp}} = \frac{C_{np}^{r_{tp}}}{C_p^{r_{tp}} + C_{np}^{r_{tp}}} \frac{S}{N} \tag{S130}$$

.

The differential equations that describe the dynamics are:

$$\begin{aligned}\frac{dS}{dt} = &- \left[\lambda^w \left(\Theta_p^w + \Theta_{np}^w\right) + \lambda^{r_t} \left(\Theta_p^{r_t} + \Theta_{np}^{r_t}\right)\right. \\ &\left. + \lambda^{r_p} \left(\Theta_p^{r_p} + \Theta_{np}^{r_p}\right) + \lambda^{r_{tp}} \left(\Theta_p^{r_{tp}} + \Theta_{np}^{r_{tp}}\right)\right]\end{aligned} \tag{S131}$$

and for the wild-type contact classes

$$\frac{dC_p^w}{dt} = \kappa \epsilon \omega \left\{\alpha_p^w \left(1 - \rho_p\right) E_p^w + \left[\left(1 - \rho_t\right) \psi + \left(1 - \psi\right)\right] \alpha_{np}^w E_{np}^w\right\} - \delta C_p^w - \lambda^w \Theta_p^w \tag{S132}$$
$$\begin{aligned}\frac{dC_{np}^w}{dt} = &\kappa \left(1 - \epsilon\right) \omega \left\{\alpha_p^w \left(1 - \rho_p\right) E_p^w + \left[\left(1 - \rho_t\right) \psi + \left(1 - \psi\right)\right] \alpha_{np}^w E_{np}^w\right\} \\ &+ \kappa \omega \left\{\left(1 - \alpha_p^w\right) \left(1 - \rho_p\right) E_p^w + \left(1 - \alpha_{np}^w\right) E_{np}^w\right\} - \delta C_{np}^w - \lambda^w \Theta_{np}^w\end{aligned} \tag{S133}$$

and for the prophylaxis-strain resistant contact classes

$$\frac{dC_p^{r_p}}{dt} = \kappa\epsilon\omega\left\{\alpha^{r_p}E_p^{r_p} + \left[(1-\rho_t)\psi + (1-\psi)\right]\alpha^{r_p}E_{np}^{r_p} + \alpha^{r_p}\rho_p E_p^w\right\} - \delta C_p^{r_p} - \lambda^{r_p}\Theta_p^{r_p} \quad \text{(S134)}$$

$$\begin{aligned}\frac{dC_{np}^{r_p}}{dt} &= \kappa(1-\epsilon)\omega\left\{\alpha^{r_p}E_p^{r_p} + \left[(1-\rho_t)\psi + (1-\psi)\right]\alpha^{r_p}E_{np}^{r_p} + \alpha^{r_p}\rho_p E_p^w\right\} \\ &\quad + \kappa\omega\left\{(1-\alpha^{r_p})E_p^{r_p} + (1-\alpha^{r_p})E_{np}^{r_p} + (1-\alpha^{r_p})\rho_p E_p^w\right\} \\ &\quad - \delta C_{np}^{r_p} - \lambda^{r_p}\Theta_{np}^{r_p}\end{aligned} \quad \text{(S135)}$$

and for the treatment-strain resistant contact classes

$$\frac{dC_p^{r_t}}{dt} = \kappa\epsilon\omega\left\{\alpha_p^{r_t}(1-\rho_p)E_p^{r_t} + \alpha_{np}^{r_t}E_{np}^{r_t} + \alpha_{np}^w\rho_t\psi E_{np}^w\right\} - \delta C_p^{r_t} - \lambda^{r_t}\Theta_p^{r_t} \quad \text{(S136)}$$

$$\begin{aligned}\frac{dC_{np}^{r_t}}{dt} &= \kappa(1-\epsilon)\omega\left\{\alpha_p^{r_t}(1-\rho_p)E_p^{r_t} + \alpha_{np}^{r_t}E_{np}^{r_t} + \alpha_{np}^w\rho_t\psi E_{np}^w\right\} \\ &\quad + \kappa\omega\left\{\left(1-\alpha_p^{r_t}\right)(1-\rho_p)E_p^{r_t} + \left(1-\alpha_{np}^{r_t}\right)E_{np}^{r_t}\right\} - \delta C_{np}^{r_t} - \lambda^{r_t}\Theta_{np}^{r_t}\end{aligned} \quad \text{(S137)}$$

and for the multi-drug resistant contact classes

$$\frac{dC_p^{r_{tp}}}{dt} = \kappa\epsilon\omega\left\{\alpha^{r_{tp}}\left(E_p^{r_{tp}} + E_{np}^{r_{tp}}\right) + \alpha^{r_{tp}}\rho_p E_p^{r_t} + \alpha^{r_p}\rho_t\psi E_{np}^{r_p}\right\} - \delta C_p^{r_{tp}} - \lambda^{r_{tp}}\Theta_p^{r_{tp}} \quad \text{(S138)}$$

$$\begin{aligned}\frac{dC_{np}^{r_{tp}}}{dt} &= \kappa(1-\epsilon)\omega\left\{\alpha^{r_{tp}}\left(E_p^{r_{tp}} + E_{np}^{r_{tp}}\right) + \alpha^{r_{tp}}\rho_p E_p^{r_t} + \alpha^{r_p}\rho_t\psi E_{np}^{r_p}\right\} \\ &\quad + \kappa\omega\left\{(1-\alpha^{r_{tp}})\left(E_p^{r_{tp}} + E_{np}^{r_{tp}}\right) + (1-\alpha^{r_{tp}})\rho_p E_p^{r_t}\right\} \\ &\quad - \delta C_{np}^{r_{tp}} - \lambda^{r_{tp}}\Theta_{np}^{r_{tp}}\end{aligned} \quad \text{(S139)}$$

and, for $x \in \{p, np\}$, $y \in \{w, r_p, r_t, r_{tp}\}$,

$$\frac{dE_x^y}{dt} = \lambda^y\Theta_x^y - \omega E_x^y \quad \text{(S140)}$$

and, for symptomatic infectious states

$$\frac{dI_p^w}{dt} = \alpha_p^w\left(1-\rho_p\right)\omega E_p^w - \gamma_I I_p^w \tag{S141}$$

$$\frac{dI_{np,t}^w}{dt} = \alpha_{np}^w\left(1-\rho_t\right)\psi\omega E_{np}^w - \gamma_I I_{np,t}^w \tag{S142}$$

$$\frac{dI_{np,nt}^w}{dt} = \alpha_{np}^w\left(1-\psi\right)\omega E_{np}^w - \gamma_I I_{np,nt}^w \tag{S143}$$

$$\frac{dI_p^{r_p}}{dt} = \alpha^{r_p}\rho_p\omega E_p^w + \alpha^{r_p}\omega E_p^{r_p} - \gamma_I I_p^{r_p} \tag{S144}$$

$$\frac{dI_{np,t}^{r_p}}{dt} = \alpha^{r_p}\left(1-\rho_t\right)\psi\omega E_{np}^{r_p} - \gamma_I I_{np,t}^{r_p} \tag{S145}$$

$$\frac{dI_{np,nt}^{r_p}}{dt} = \alpha^{r_p}\left(1-\psi\right)\omega E_{np}^{r_p} - \gamma_I I_{np,nt}^{r_p} \tag{S146}$$

$$\frac{dI_p^{r_t}}{dt} = \alpha_p^{r_t}\left(1-\rho_p\right)\omega E_p^{r_t} - \gamma_I I_p^{r_t} \tag{S147}$$

$$\frac{dI_{np}^{r_t}}{dt} = \alpha_{np}^w\rho_t\psi\omega E_{np}^w + \alpha_{np}^{r_t}\omega E_{np}^{r_t} - \gamma_I I_{np}^{r_t} \tag{S148}$$

$$\frac{dI_p^{r_{tp}}}{dt} = \alpha^{r_{tp}}\rho_p\omega E_p^{r_t} + \alpha^{r_{tp}}\omega E_p^{r_{tp}} - \gamma_I I_p^{r_{tp}} \tag{S149}$$

$$\frac{dI_{np}^{r_{tp}}}{dt} = \alpha^{r_p}\rho_t\psi\omega E_{np}^{r_p} + \alpha^{r_{tp}}\omega E_{np}^{r_{tp}} - \gamma_I I_{np}^{r_{tp}} \tag{S150}$$

and, for asymptomatic infectious states

$$\frac{dA_p^w}{dt} = \left(1-\alpha_p^w\right)\left(1-\rho_p\right)\omega E_p^w - \gamma_A A_p^w \tag{S151}$$

$$\frac{dA_{np}^w}{dt} = \left(1-\alpha_{np}^w\right)\omega E_{np}^w - \gamma_A A_{np}^w \tag{S152}$$

$$\frac{dA_p^{r_p}}{dt} = \left(1-\alpha^{r_p}\right)\rho_p\omega E_p^w + \left(1-\alpha^{r_p}\right)\omega E_p^{r_p} - \gamma_A A_p^{r_p} \tag{S153}$$

$$\frac{dA_{np}^{r_p}}{dt} = \left(1-\alpha^{r_p}\right)\omega E_{np}^{r_p} - \gamma_A A_{np}^{r_p} \tag{S154}$$

$$\frac{dA_p^{r_t}}{dt} = \left(1-\alpha_p^{r_t}\right)\left(1-\rho_p\right)\omega E_p^{r_t} - \gamma_A A_p^{r_t} \tag{S155}$$

$$\frac{dA_{np}^{r_t}}{dt} = \left(1-\alpha_{np}^{r_t}\right)\omega E_{np}^{r_t} - \gamma_A A_{np}^{r_t} \tag{S156}$$

$$\frac{dA_p^{r_{tp}}}{dt} = \left(1-\alpha^{r_{tp}}\right)\rho_p\omega E_p^{r_t} + \left(1-\alpha^{r_{tp}}\right)\omega E_p^{r_{tp}} - \gamma_A A_p^{r_{tp}} \tag{S157}$$

$$\frac{dA_{np}^{r_{tp}}}{dt} = \left(1-\alpha^{r_{tp}}\right)\omega E_{np}^{r_{tp}} - \gamma_A A_{np}^{r_{tp}} \tag{S158}$$

and, for $x \in \{p, np\}$, $y \in \{w, r_p, r_t, r_{tp}\}$

$$\frac{dR_{Ix}^y}{dt} = \gamma_I I_x^y \tag{S159}$$

$$\frac{dR_{Ax}^y}{dt} = \gamma_A A_x^y \tag{S160}$$

and finally, for the depletion of the finite stockpiles of AV drugs,

$$\frac{dO_p}{dt} = -\kappa\epsilon\omega\left[\alpha_p^w E_p^w + \alpha_{np}^w E_{np}^w + \alpha_p^{rt} E_p^{rt} + \alpha_{np}^{rt} E_{np}^{rt} + \alpha^{r_p}\left(E_p^{r_p} + E_{np}^{r_p}\right)\right. \\ \left. + \alpha^{r_{tp}}\left(E_p^{r_{tp}} + E_{np}^{r_{tp}}\right)\right] \tag{S161}$$

$$\frac{dO_t}{dt} = -\psi\omega\left[\alpha_{np}^w E_{np}^w + \alpha_{np}^{rt} E_{np}^{rt} + \alpha^{r_p} E_{np}^{r_p} + \alpha^{r_{tp}} E_{np}^{r_{tp}}\right]. \tag{S162}$$

When the stockpile of AV drugs for prophylaxis, O_p, or treatment, O_t, runs out that form of intervention is no longer possible. Within the simulations, we set ϵ or ψ to zero as appropriate from that point onwards.

R_0 is, again, the maximum of four eigenvalues (one for each of the strains):

$$R_0 = \frac{\beta}{\gamma} \times \max \begin{cases} \left(\alpha_p^w + \left(1-\alpha_p^w\right)\chi\right)\left(1-\rho_p\right) e_s e_i K^w \\ \qquad + \left(\alpha_{np}^w\left[e_t\left(1-\rho_t\right)\psi + \left(1-\psi\right)\right] + \left(1-\alpha_{np}^w\right)\chi\right)\left(1-K^w\right), \\ \phi^{r_p}\left\{\left(\alpha^{r_p} + \left(1-\alpha^{r_p}\right)\chi\right)K^{r_p} \right. \\ \qquad \left. + \left(\alpha^{r_p}\left[e_t\left(1-\rho_t\right)\psi + \left(1-\psi\right)\right] + \left(1-\alpha^{r_p}\right)\chi\right)\left(1-K^{r_p}\right)\right\}, \\ \phi^{r_t}\left\{\left(\alpha_p^{rt} + \left(1-\alpha_p^{rt}\right)\chi\right)\left(1-\rho_p\right) e_s e_i K^{r_t} \right. \\ \qquad \left. + \left(\alpha_{np}^{rt} + \left(1-\alpha_{np}^{rt}\right)\chi\right)\left(1-K^{r_t}\right)\right\}, \\ \phi^{r_{tp}}\left\{\alpha^{r_{tp}} + \left(1-\alpha^{r_{tp}}\right)\chi\right\} \end{cases} \tag{S163}$$

S2 Results

We consider a number of different scenarios to demonstrate the general nature of the results presented in the main text. We also provide a sensitivity analysis for the single-drug, random allocation and treatment and prophylaxis models.

S2.1 Alternative scenarios

In this section we examine two plausible alternative scenarios where it would be expected that a successful intervention is possible:

1. The resistant strain has a higher relative fitness than $\phi = 0.8$, but a lower seeding rate. We examine a 90% fit resistant strain ($\phi = 0.9$) with a seeding rate of 10 or 100 times lower ($\rho_t = 10^{-2}$ or $\rho_t = 10^{-3}$). We keep $\rho_p = \rho_t/10$.

2. The symptomatic proportion is somewhat lower. With a fixed clinical attack rate of 40% this implies a higher R_0. We examine a scenario with $\alpha = 0.6$ and an increased (but still realistic) intervention of providing prophylaxis to 40% of contacts (up from 30% in the main text) and treatment to 50% of symptomatic cases (up from 40% in the main text).

S2.1.1 Extremely high fitness and lower seeding

We keep the symptomatic proportion unchanged at $\alpha_{np}^w = 0.7$, and the intervention unchanged at $\epsilon = 0.3$, $\psi = 0.4$. We restrict ourselves to a 90–10 stockpile. Figures S3– S5 are the equivalent of Figures 5–7 in the main text. While each strategy is less capable of controlling the epidemic compared to the main text, the treatment and prophylaxis strategy remains the most effective choice. In fact, especially in the case of lower seeding, it is more clear cut that treatment and prophylaxis is

a better strategy compared to either single-drug usage or a random allocation strategy. The reason is clear: with higher fitness ($\phi = 0.9$) it becomes even more important to delay emergence of the multi-drug resistant strain.

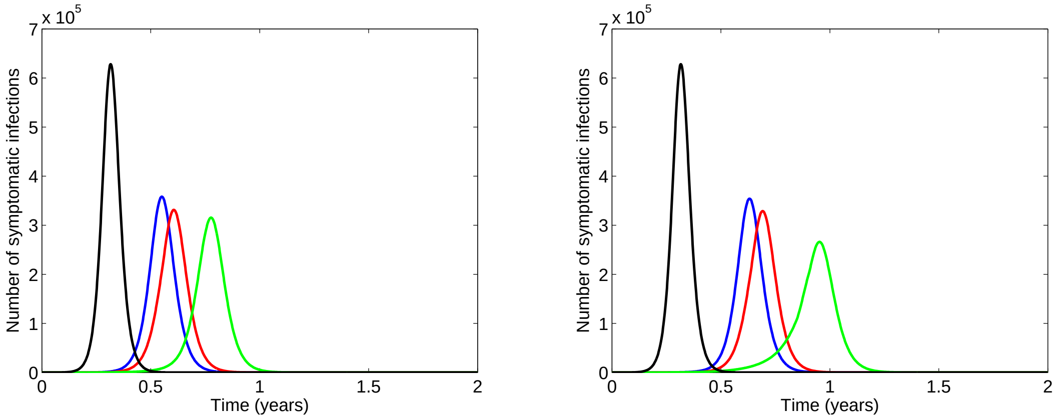

(a) (b)

Figure S3: **a.** $\rho_t = 0.01$ (one order of magnitude less than usual) **b.** $\rho_t = 0.001$ (two orders of magnitude less than usual).

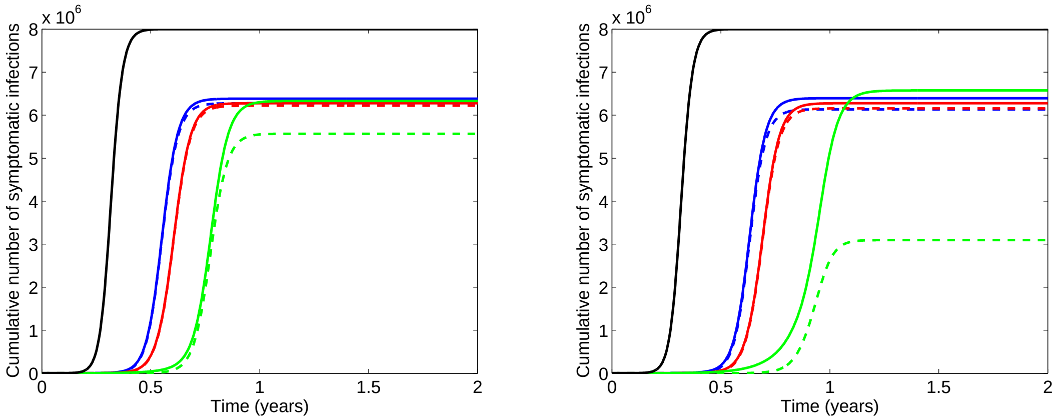

(a) (b)

Figure S4: **a.** $\rho_t = 0.01$ (one order of magnitude less than usual) **b.** $\rho_t = 0.001$ (two orders of magnitude less than usual).

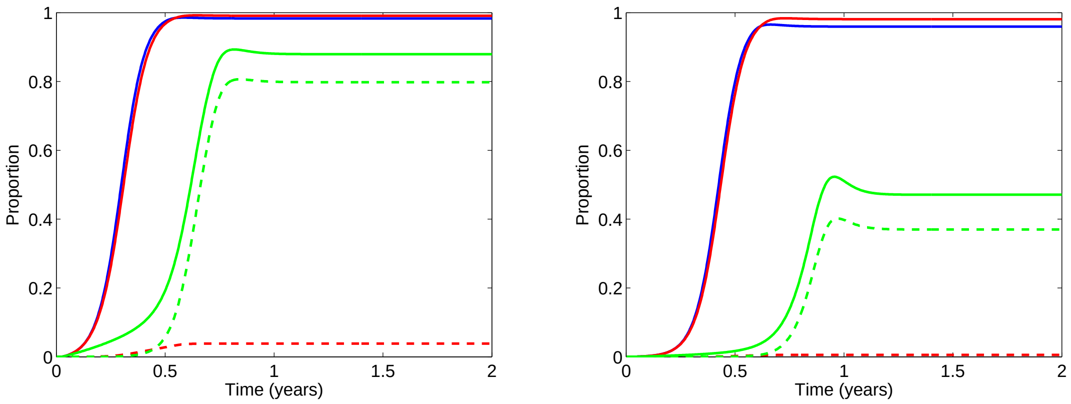

(a) (b)

Figure S5: **a.** $\rho_t = 0.01$ (one order of magnitude less than usual) **b.** $\rho_t = 0.001$ (two orders of magnitude less than usual).

S2.1.2 A reduced symptomatic proportion

Consider a scenario where $\alpha = 0.6$ ($R_0 = 1.x$). Without an increase in the intervention the impact of treatment and/or prophylaxis is minimal (see Figure 2b in the main text). Here, we examine the ability of an increased intervention to delay the onset of the epidemic. Because the baseline epidemic duration is shorter, an increased intervention can not produce delays to median infection as great as for $\alpha = 0.7$. However, a similar multiplicative increase in the time to median infection can be achieved and the different strategies (single-drug, random allocation, treatment and prophylaxis) have the same qualitative impacts. Figure S6 shows the epidemic curves, cumulative curves and cumulative resistant proportion for a 90–10 stockpile scenario.

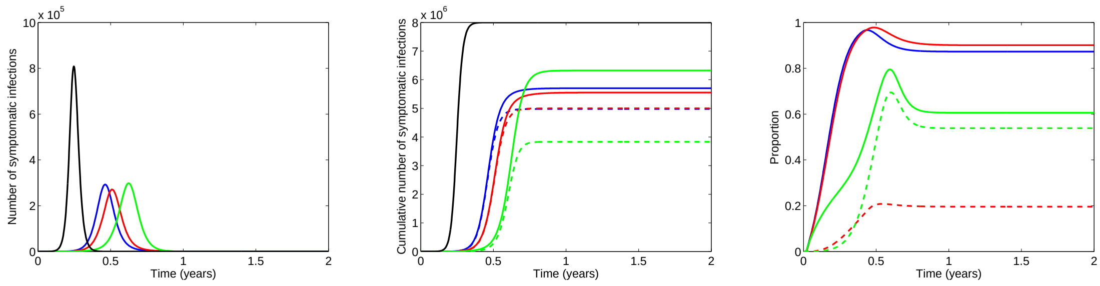

(a) (b) (c)

Figure S6: $\alpha^w_{np} = 0.6$, $\epsilon = 0.4$, $\psi = 0.5$. **a.** Epidemic curves. **b.** Cumulative curves. **c** Cumulative resistance proportion.

We explore this scenario ($\alpha = 0.6$, $\epsilon = 0.4$, $\psi = 0.5$) a little more below when performing a sensitivity analysis for multi-drug strategies.

S2.2 Sensitivity Analysis

Throughout the main text we assumed that:

1. antiviral drugs had a fixed efficacy. For treatment, we set $e_t = 0.7$ (relative infectiousness). For prophylaxis, we set $e_s = 0.5$ (relative susceptibility) and $e_i = 0.4$ (relative infectiousness if nevertheless infected).

2. resistant strain seeding was either "high" ($\rho_t = 10^{-1}$, $\rho_p = 10^{-2}$) or "low" ($\rho_t = 10^{-3}$, $\rho_p = 10^{-4}$).

3. relative transmissibility of asymptomatic infections was 50% ($\chi = 0.5$).

Here, we examine sensitivity to these parameter choices. We also compare conclusions regarding optimal strategy across these same parameter sensitivity analyses. Throughout, we restrict ourselves to a high-fitness ($\phi = 0.8$) resistant strain scenario. In the multi-drug strategy section we further restrict the analysis to high seeding only ($\rho_t = 10^{-1}$, $\rho_p = 10^{-2}$).

We perform this analysis with the symptomatic proportion fixed as in the main text ($\alpha^w_{np} = 0.7$). The intervention also remains as in the main text ($\epsilon = 0.3$, $\psi = 0.4$).

S2.2.1 Single drug model

With a single drug for treatment and prophylaxis, it is implausible that e_t and e_i vary independently. Thus, we tie e_t to e_i: as e_i varies from 0.4 to 1, e_t varies from 0.7 to 1. Figure S7 shows the single-drug model's sensitivity to variation in antiviral drug efficacy.

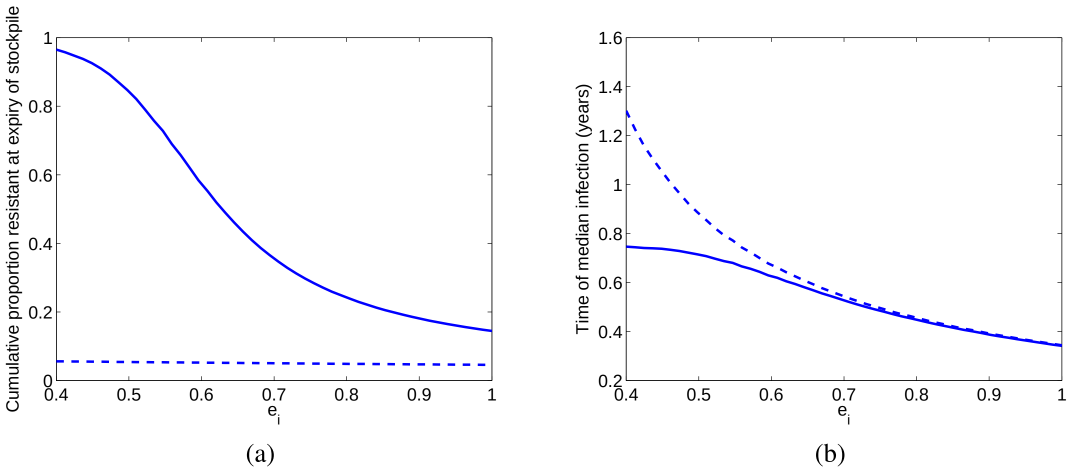

Figure S7: Impact of simultaneously varying $e_i \in [0.4, 1]$, $e_t \in [0.7, 1]$ and $e_s \in [0.5, 1]$, for high fitness ($\phi = 0.8$, solid line) and low fitness ($\phi = 0.3$, dashed line). The seeding rate is kept fixed at $\rho_t = 10^{-1}$, $\rho_p = 10^{-2}$. **a.** The unfit resistant strain (dashed line) is unable to establish itself in the population. For the fit resistant strain (solid line) the proportion of resistant infections at stockpile expiry increases as ability to reduce transmission increases (e_i, e_t and e_s getting smaller). **b.** The unfit resistant strain (dashed line) always leads to a longer time to median infection than the fit resistant strain (solid line). For the unfit strain, improving AV transmission reduction (e_i getting smaller) increases the time to median infection. For the fit resistant strain, as the AV intervention becomes effective enough to lead to a resistant-strain dominated epidemic we find the time to median infection flattens out. The properties of the AV intervention have little influence on an epidemic dominated by resistant virus.

Figure S8 shows the single-drug model's sensitivity to variation in the seeding rate of the resistant strain. We always keep $\rho_p = \rho_t/10$.

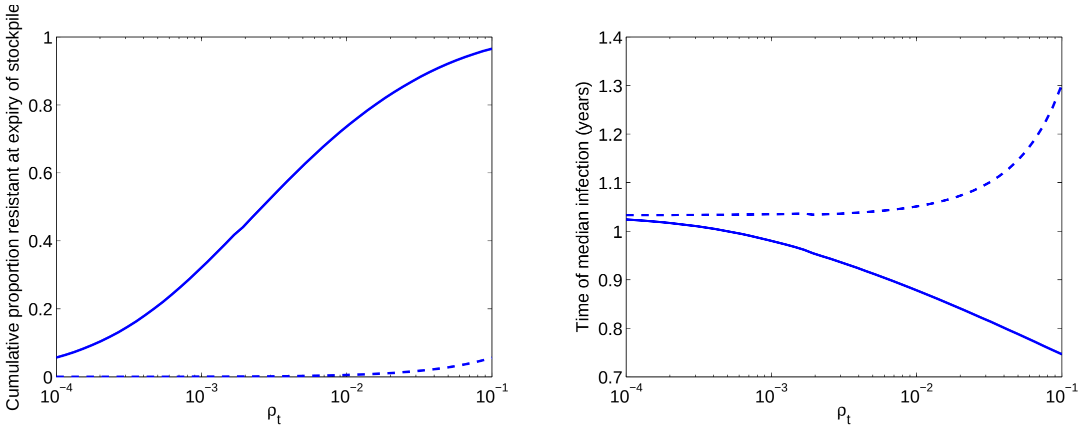

(a) (b)

Figure S8: Impact of varying ρ_t and ρ_p (logscale) for ϕ above the threshold (solid line) and ϕ below the threshold (dashed line). **a.** Above the threshold, increasing the seeding rate increases the proportion resistant. Below the threshold, the proportion resistant is essentially independent of ρ_t and ρ_p. **b.** Time to median infection decreases as seeding rate increases above the threshold and increases above the threshold (the "immunising" effect).

Figure S9 shows the single-drug model's sensitivity to variaiton in the assumed relative transmissibility of asymptomatic infections.

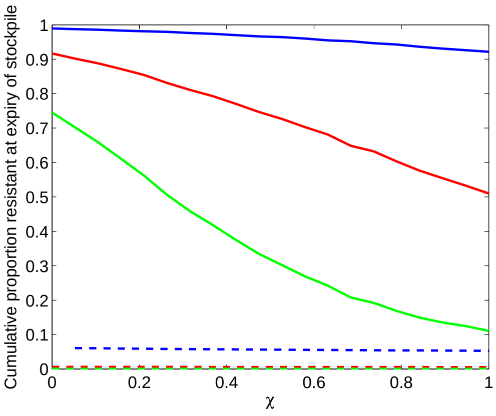

(a)

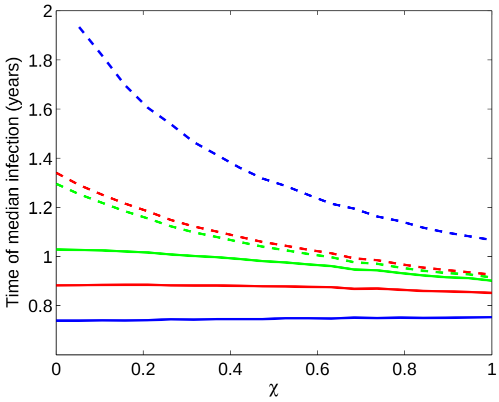

(b)

Figure S9: Impact of varying χ. Colour is seeding rate ($\rho_t = 0.1$, $\rho_p = 0.01$ (blue), $\rho_t = 0.01$, $\rho_p = 0.001$ (red), $\rho_t = 0.001$, $\rho_p = 0.0001$ (green)). Solid lines are high fitness ($\phi = 0.8$), dashed lines are low fitness ($\phi = 0.3$). **a.** For low fitness strains there is no ability to generate significant proportions of resistance. For high fitness strains, and for high seeding (blue) we have a resistant-strain dominated epidemic. Therefore, relative transmissibility of asymptomatics is not important. For lower seeding (red and green), the epidemic is a mixture of wild-type and resistant-type strains and thus, as χ increases and the wild-type is more able to continue transmitting, the proportion resistant is reduced. **b.** For transmissible resistant strains (solid lines) we generally have significant proportions resistant so the time to median infection is fairly independent of χ. For untransmissible resistant strains (dotted lines) the time to median infection is reduced as asymptomatics are more capable of transmitting.

S2.2.2 Random allocation model

In a random allocation strategy, both drugs 1 and 2 are used for treatment and prophylaxis. Therefore, for each drug we must tie e_i, e_t and e_s as in the single drug strategy. However, we can vary the efficacy of the two drugs. For a fixed fitness and seeding ($\phi = 0.8$, $\rho_t = 10^{-1}$, $\rho_p = 10^{-2}$), we vary e_{i1} and e_{i2} independently. e_{t1} and e_{s1} are locked to e_{i1} and e_{t2} and e_{s2} are locked to e_{i2} as in the single drug strategy sensitivity analysis. Figure S10 shows the random allocation model's sensitivity to variation in antiviral drug efficacy for a 90–10 stockpile split. Figure S11 shows the result for a 50–50 stockpile split.

For the 90–10 split, we see, unsurprisingly, little sensitivity to the properties of drug 2. For the 50–50 split the system is symmetric in drug 1 and drug 2.

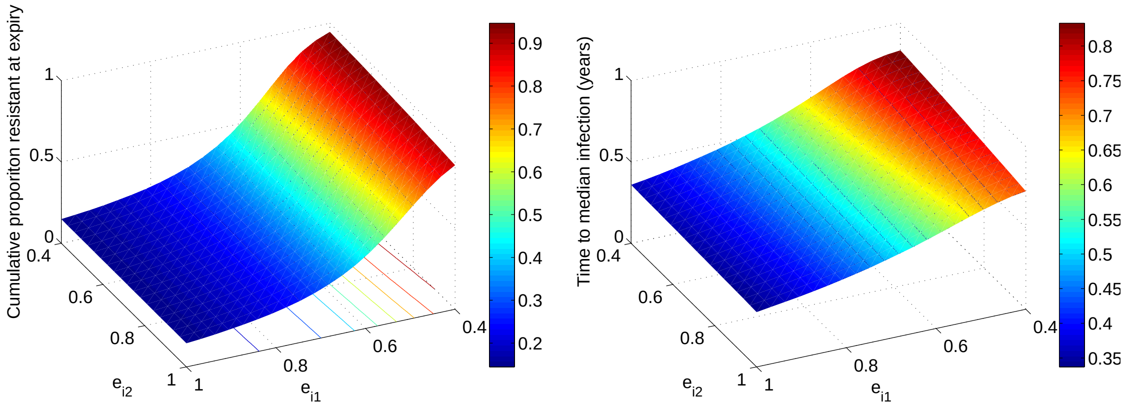

(a) (b)

Figure S10: Random allocation sensitivity to e_{i1} and e_{i2}. e_{t1} and e_{t2} vary in the interval $[0.7, 1]$ and e_{s1} and e_{s2} vary in the interval $[0.5, 1]$ as e_{i1} and e_{i2} vary in the interval $[0.4, 1]$. The stockpile split is 90–10. **a.** Cumulative proportion resistant at expiry of stockpile. **b.** Time to median infection.

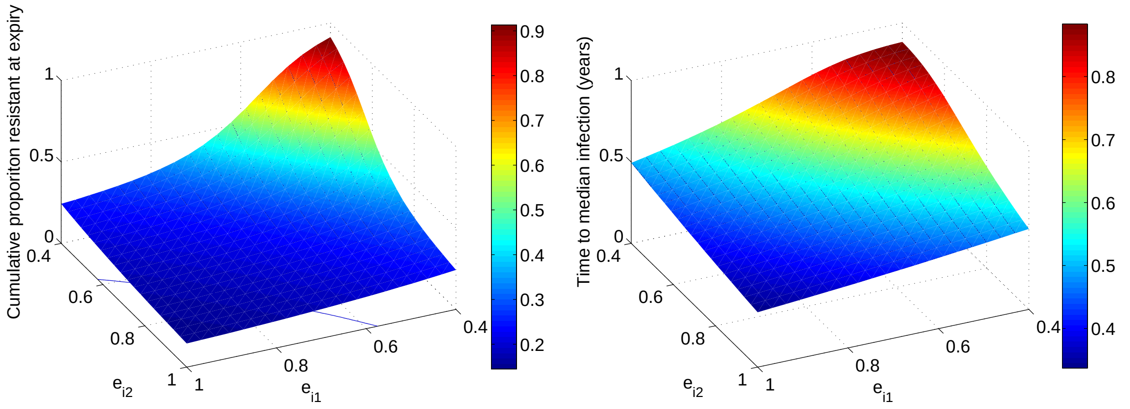

(a) (b)

Figure S11: Details as in Figure S11 but for a 50–50 stockpile split. **a.** Cumulative proportion resistant at expiry of stockpile. **b.** Time to median infection.

Figures S12 and S13 show the random allocation model's sensitivity to variation in the assumed relative transmissibility of asymptomatic infections with a 90–10 and 50–50 stockpile split respectively. We show two scenarios: as in the main text ($\alpha_{np}^{w} = 0.7$, $\epsilon = 0.3$, $\psi = 0.4$, blue) and the alternative scenario presented above in Section S2.1.2 ($\alpha_{np}^{w} = 0.6$, $\epsilon = 0.4$, $\psi = 0.5$, red).

For the 90-10 stockpile split, most resistance (that is, more than half) is single-drug resistance. The results are largely insensitive to χ While the proportion resistant at stockpile expiry is similar for the $\alpha = 0.6$ scenario (compared to the $\alpha = 0.7$ scenario), the time to median infection is much shorter. This reflects the naturally faster dynamics due to the higher R_0.

For a 50–50 stockpile, fewer of the infections are resistant in total compared to in the 90–10 case, but of those that are, multi-strain resistance is dominant.

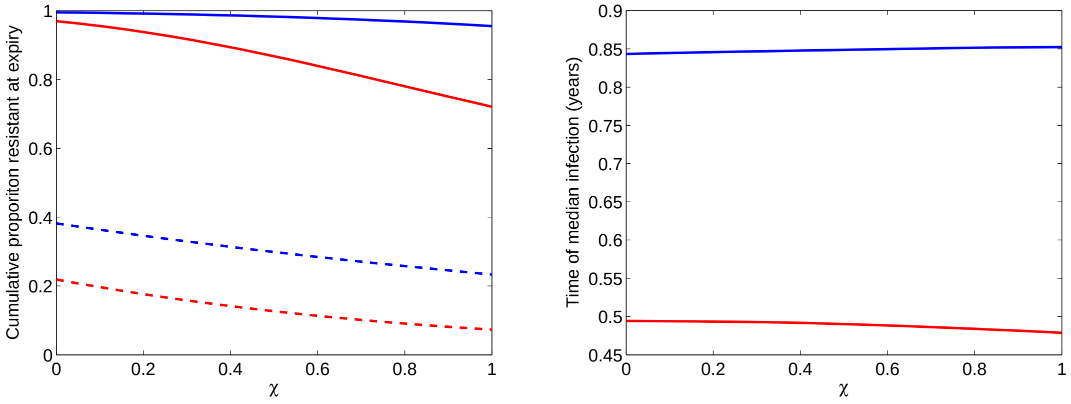

(a) (b)

Figure S12: The main text scenario is in blue ($\alpha_{np}^{w} = 0.7$, $\epsilon = 0.3$, $\psi = 0.4$). The alternative scenario is in red ($\alpha_{np}^{w} = 0.6$, $\epsilon = 0.4$, $\psi = 0.5$). The stockpile split is 90–10. **a.** Solid lines are total proportion resistant. Dashed lines are the proportion of all infections that are multi-drug resistant. **b.** Solid lines are the time of median infection.

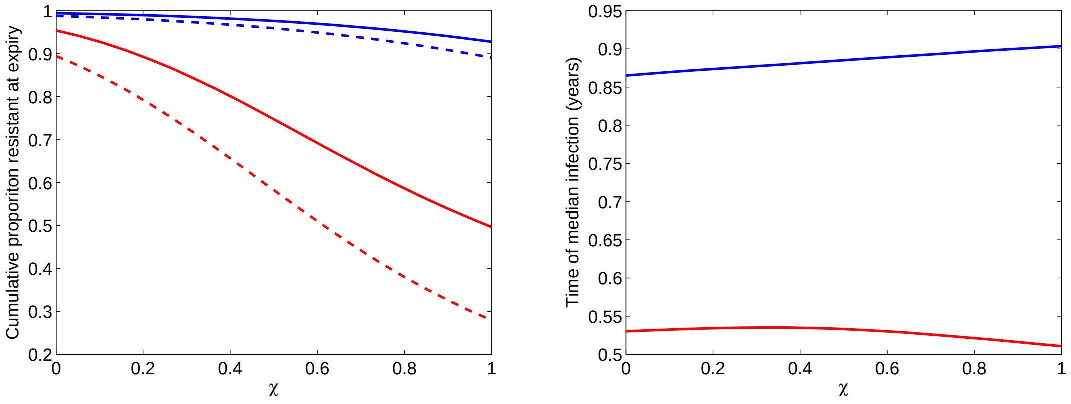

(a) (b)

Figure S13: Details as in Figure S12 but for a 50–50 stockpile.

S2.2.3 Treatment and Prophylaxis model

In a treatment and prophylaxis strategy we have one drug used solely for treatment and the other solely for prophylaxis. It follows that we examine sensitivity to antiviral drug efficacy in the (e_i, e_t)-plane. We tie e_s to e_i as in the single-drug sensitivity analysis. Figure S14 shows the proportion resistant at stockpile expiry and the time of median infection as a function of e_i and e_t.

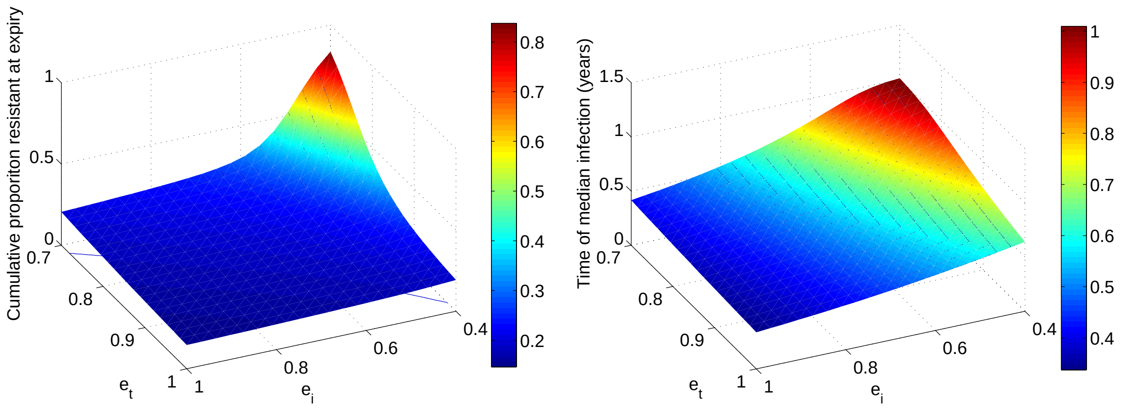

(a) (b)

Figure S14: Treatment and prophylaxis drugs strategy's sensitivity to e_i and e_t. The susceptibility factor is fixed at $e_s = 0.5$. **a.** Cumulative proportion resistant at expiry of stockpile. **b.** Time to median infection.

Figure S15 shows the variation in output as a function of relative transmissibility of asymptomatic infections, for both the main text scenario ($\alpha = 0.7$, blue) and the alternative scenario considered earlier in this document ($\alpha = 0.6$, red).

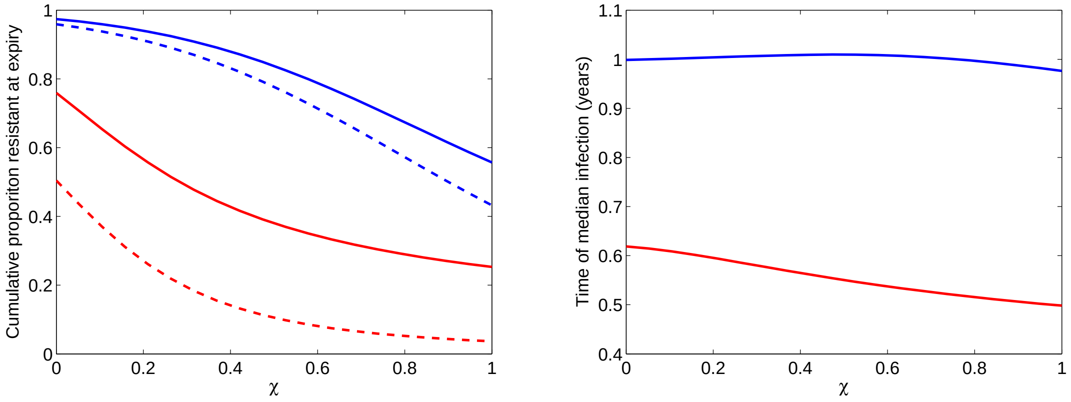

(a) (b)

Figure S15: Details as in Figure S12 but for the treatment and prophylaxis strategy.

S2.2.4 Comparing models

We have explored the sensitivity of each model to key parameter assignments. Here, as in the main text, we make a comparison between models.

A comparison of Figures S12 and S15 demonstrates that across the range of χ values considered, the treatment and prophylaxis strategy provides better outcomes, in terms of both proportion of infections resistant and time of median infection, than does the random allocation strategy. The single-drug strategy result (Figure S9, solid blue line) is inferior to both multi-drug strategies.

To make a comparison in terms of sensitivity to antiviral drug effectiveness is more difficult. If there is a large asymmetry in the effectiveness of the two drugs, the treatment and prophylaxis strategy can yield a poorer result compared to the random allocation strategy.

Consider a situation where the 10% stockpile (drug 2) turns out to be ineffective ($e_t \to 1$, $e_{i2} \to 1$, $e_{t2} \to 1$). For the random allocation strategy, this is of little consequence. The 90% stockpile (drug 1) is used for both treatment and prophylaxis and the result is essentially that given by a single-drug strategy. For the treatment and prophylaxis strategy however, we essentially have a *prophylaxis only* single-drug strategy. Our previous work (McCaw and McVernon, 2007) has shown that a "synergistic" effect is observed when effective treatment (low e_t) is introduced in a population receiving prophylaxis. Thereby, for efficacious drug 1 (low e_i, e_{i1} and e_{t1}) and poor drug 2 (high e_t, e_{i2} and e_{t2}) in Figure S16b we find that a random allocation strategy provides a longer time to median infection than does a treatment and prophylaxis strategy.

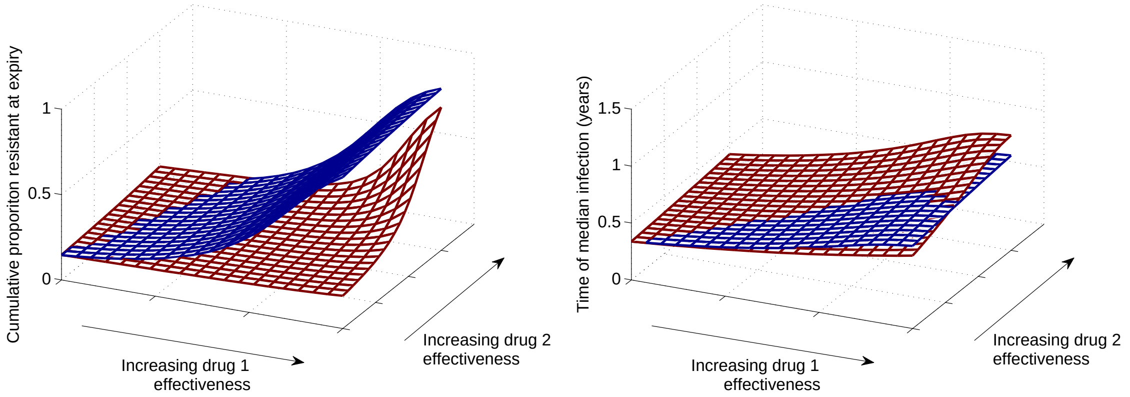

(a) (b)

Figure S16: For a 90–10 stockpile split, we compare the random allocation (blue) and treatment and prophylaxis (red) models. The two surfaces are those already presented in Figures S10 and S14. For the blue surface, e_{i1} and e_{t1} vary on the drug 1 axis, and e_{i2} and e_{t2} on the drug 2 axis. For the red surface, e_i varies on the drug 1 axis and e_t varies on the drug 2 axis. The impact on susceptibility of each drug is kept fixed throughout at $e_s = 0.5$ for both strategies. If we also tie e_s to e_i (for drug 1 in the treatment and prophylaxis model, and for both drugs in the random allocation model) we find a qualitatively similar result. **a.** Cumulative proportion resistant at stockpile expiry. The random allocation surface lies above the treatment and prophylaxis surface for most parameter values. **b.** Time of median infection. For the most part, the treatment and prophylaxis strategy provides a longer time to median infection. Only when drug 2 (the 10% drug) is ineffective and drug 1 is effective does the random allocation model provide a longer time to median infection.

A Multi-drug model construction

This technical appendix provides the diagrams and R_0 calculations for the multi-drug models.

A.1 Random allocation model

The possible flows are shown in the following figures.

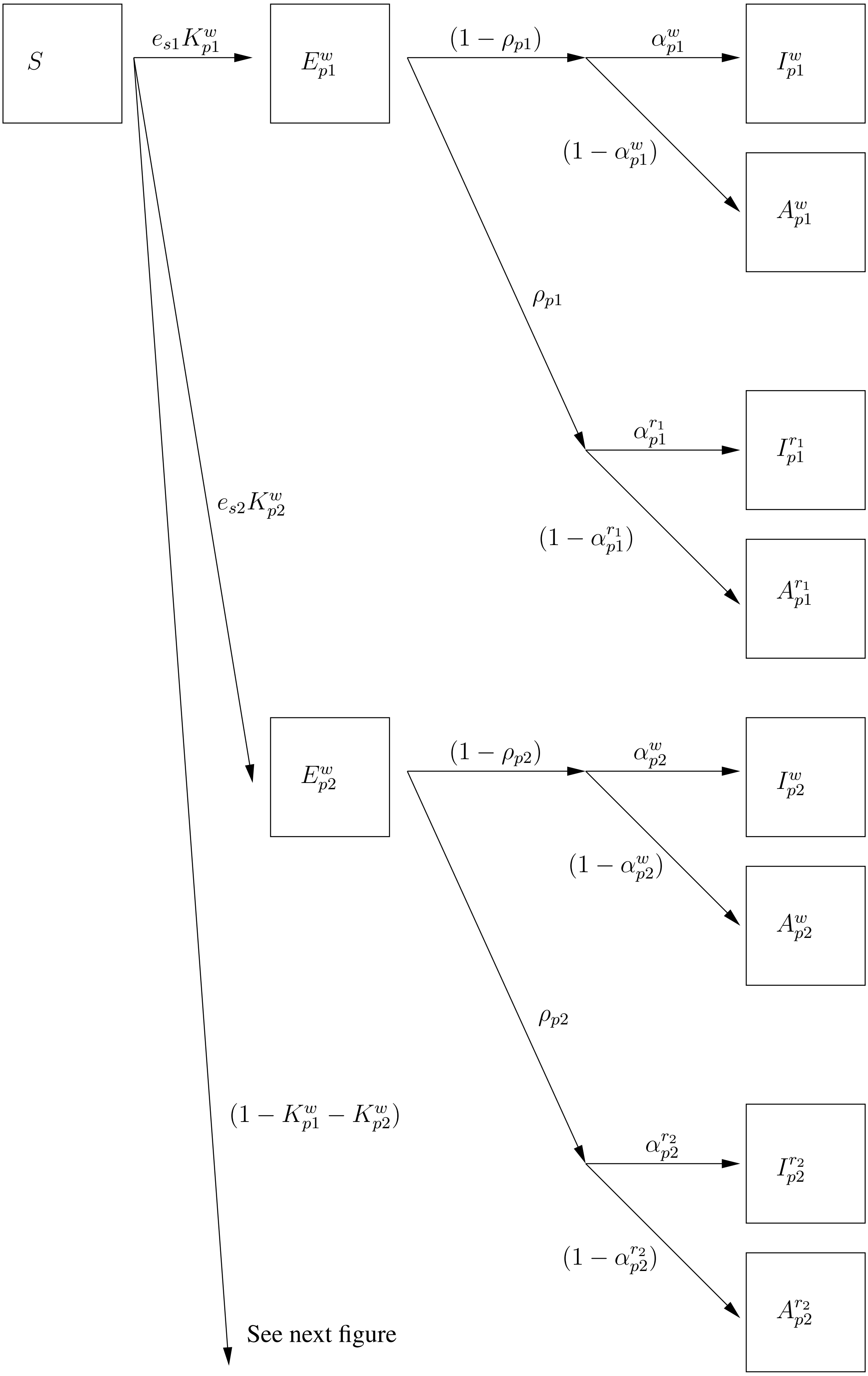

Figure S17: Tree diagram for wild strain exposures (prophylaxis part)

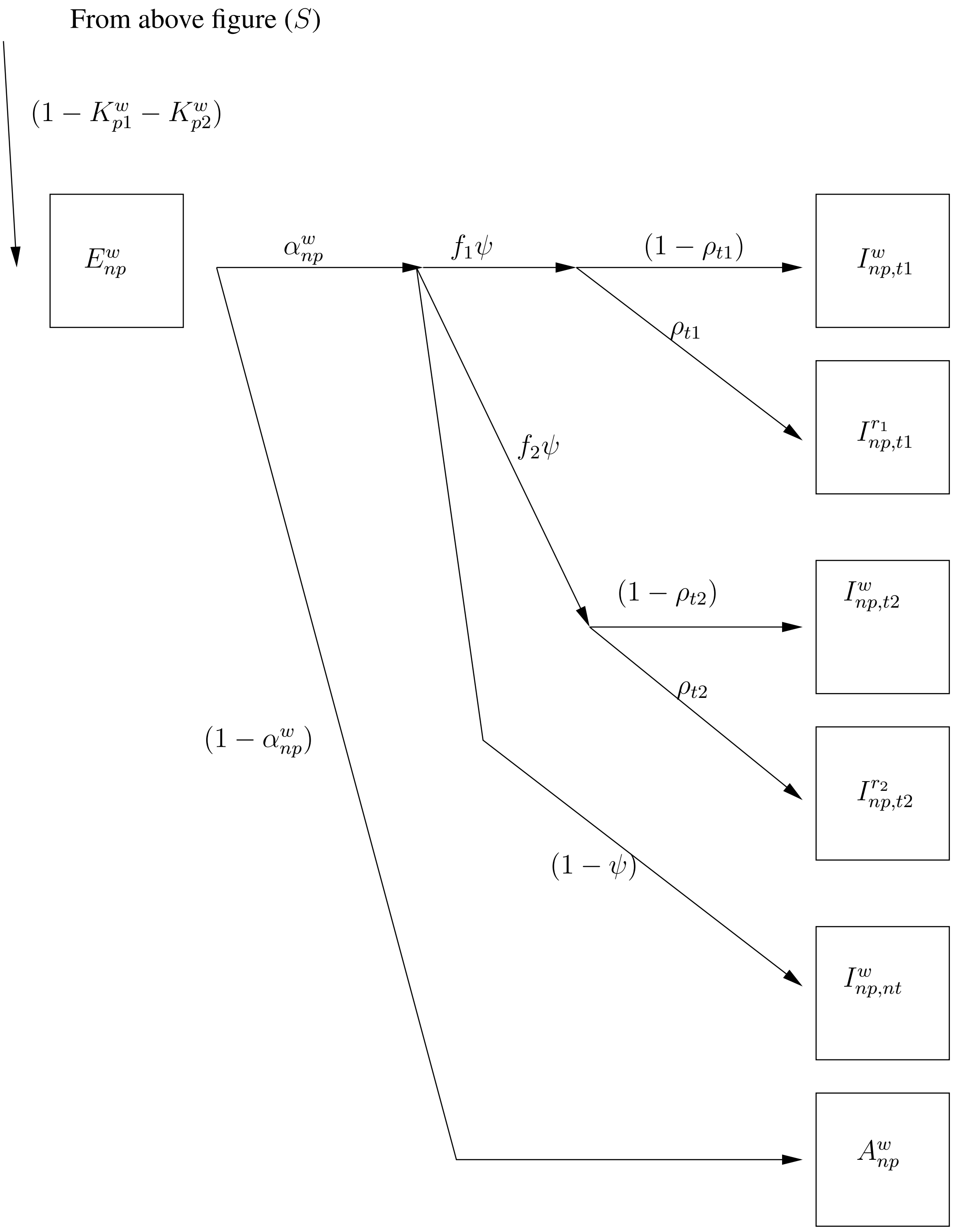

Figure S18: Tree diagram for wild strain exposures (treatment part)

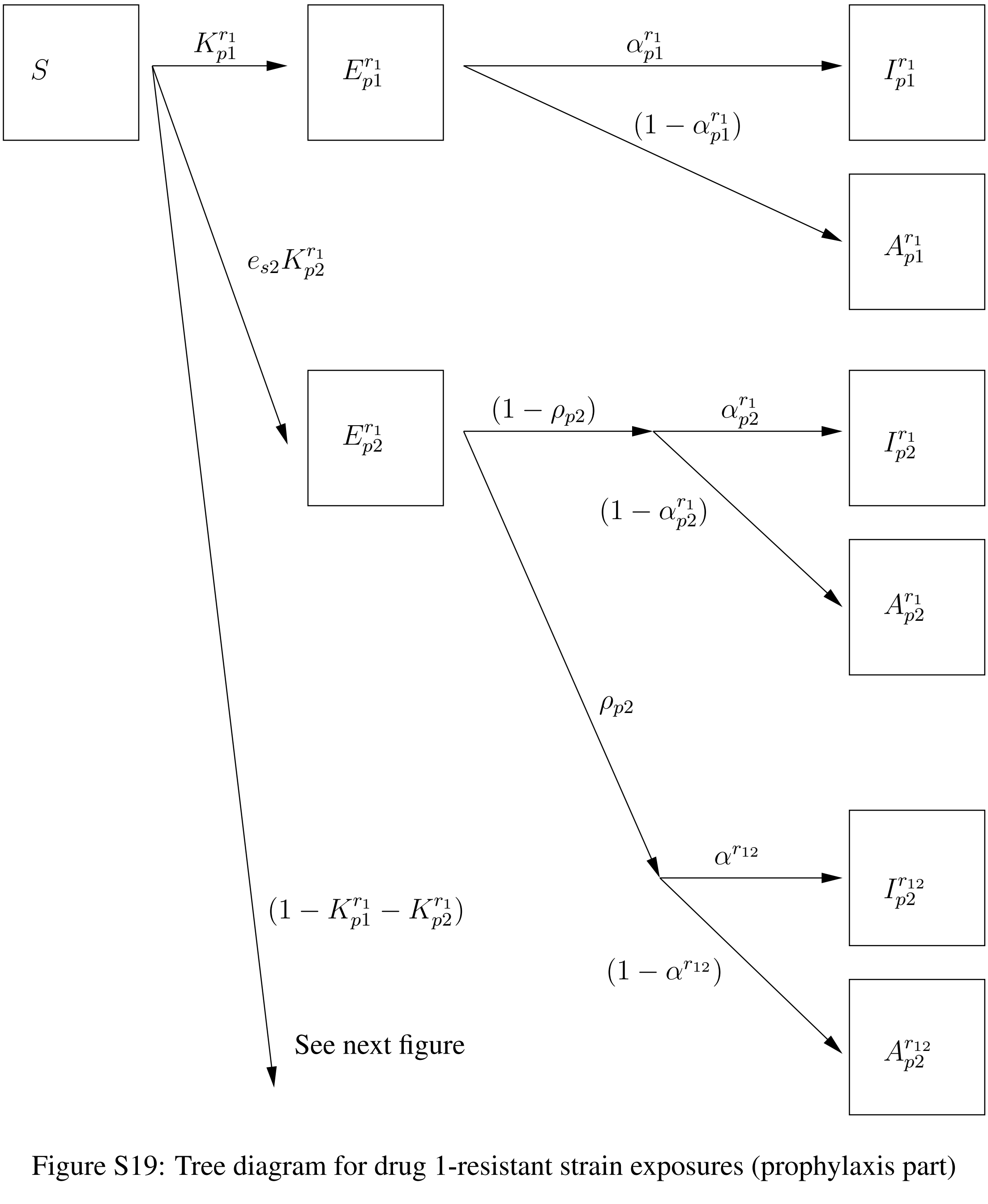

Figure S19: Tree diagram for drug 1-resistant strain exposures (prophylaxis part)

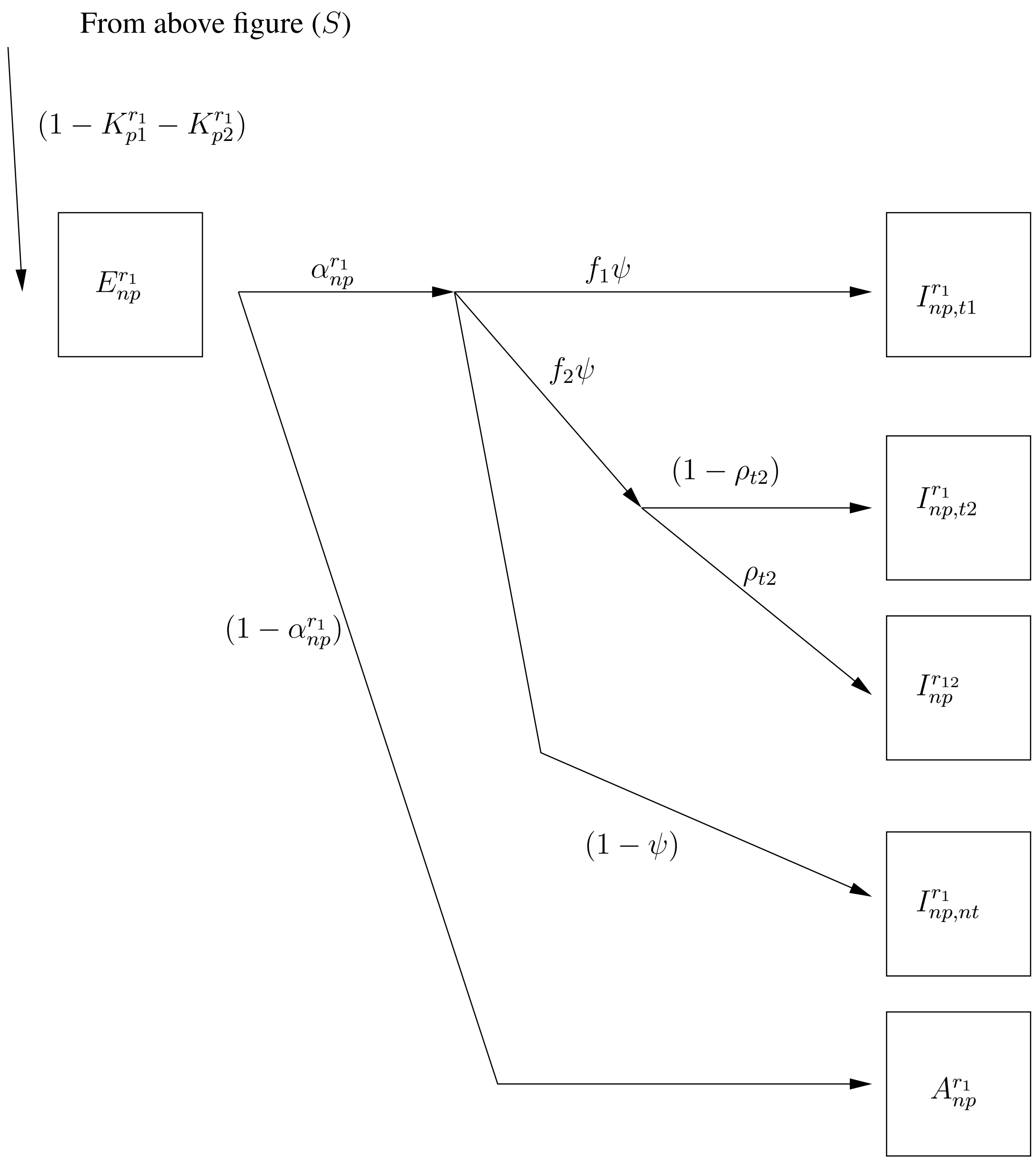

Figure S20: Tree diagram for drug 1-resistant strain exposures (treatment part)

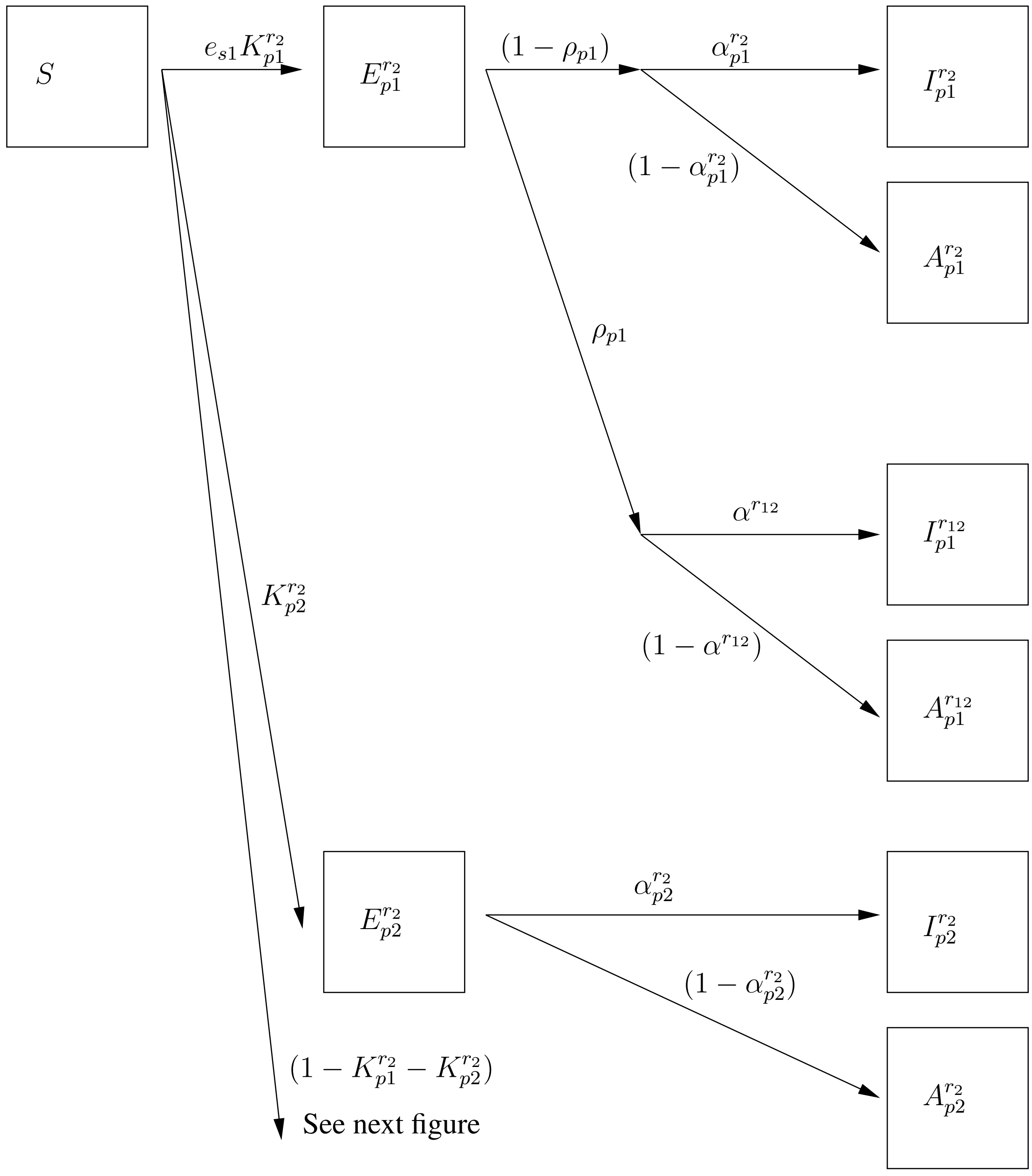

Figure S21: Tree diagram for drug 2-resistant strain exposures (prophylaxis part)

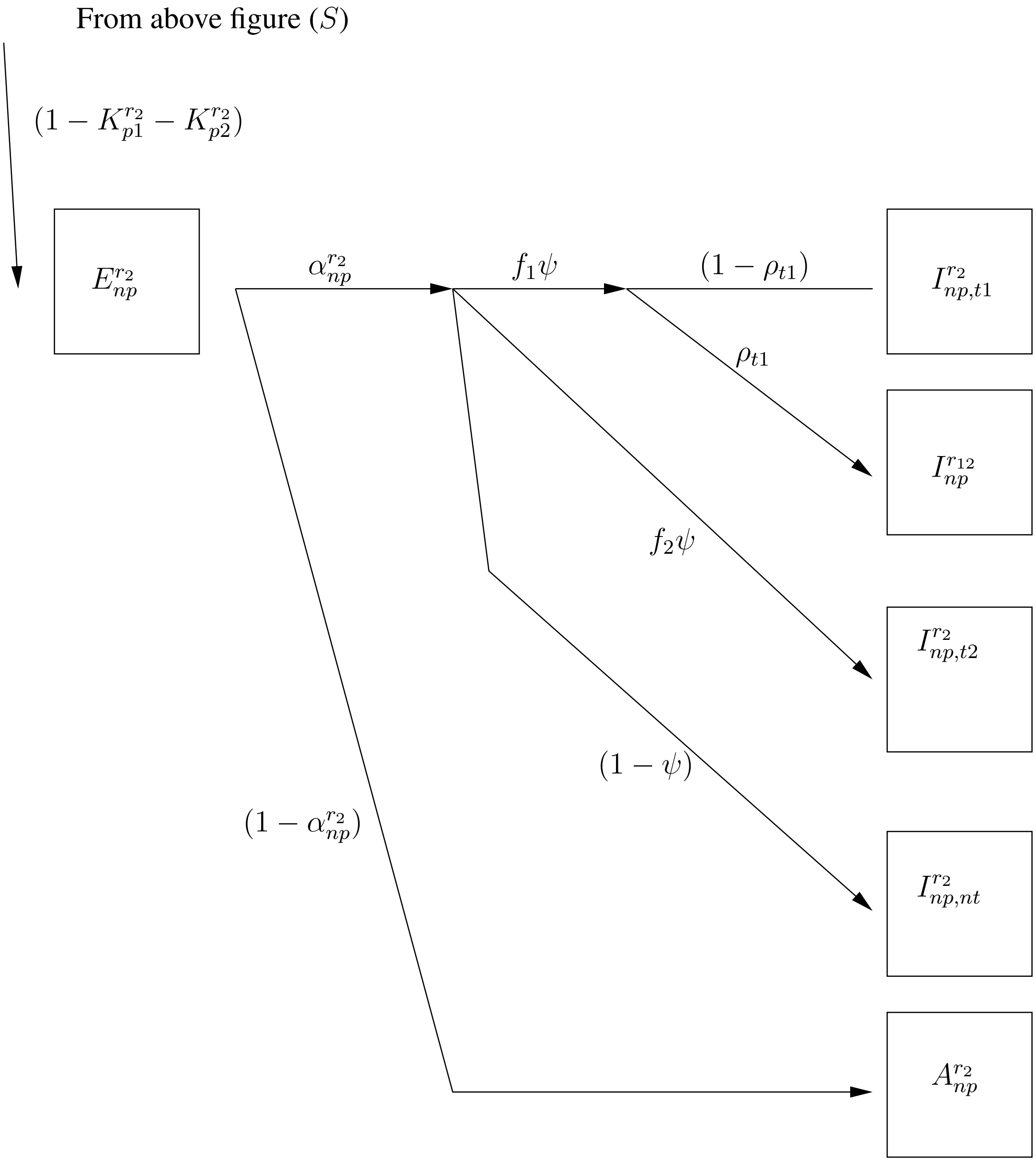

Figure S22: Tree diagram for drug 2-resistant strain exposures (treatment part)

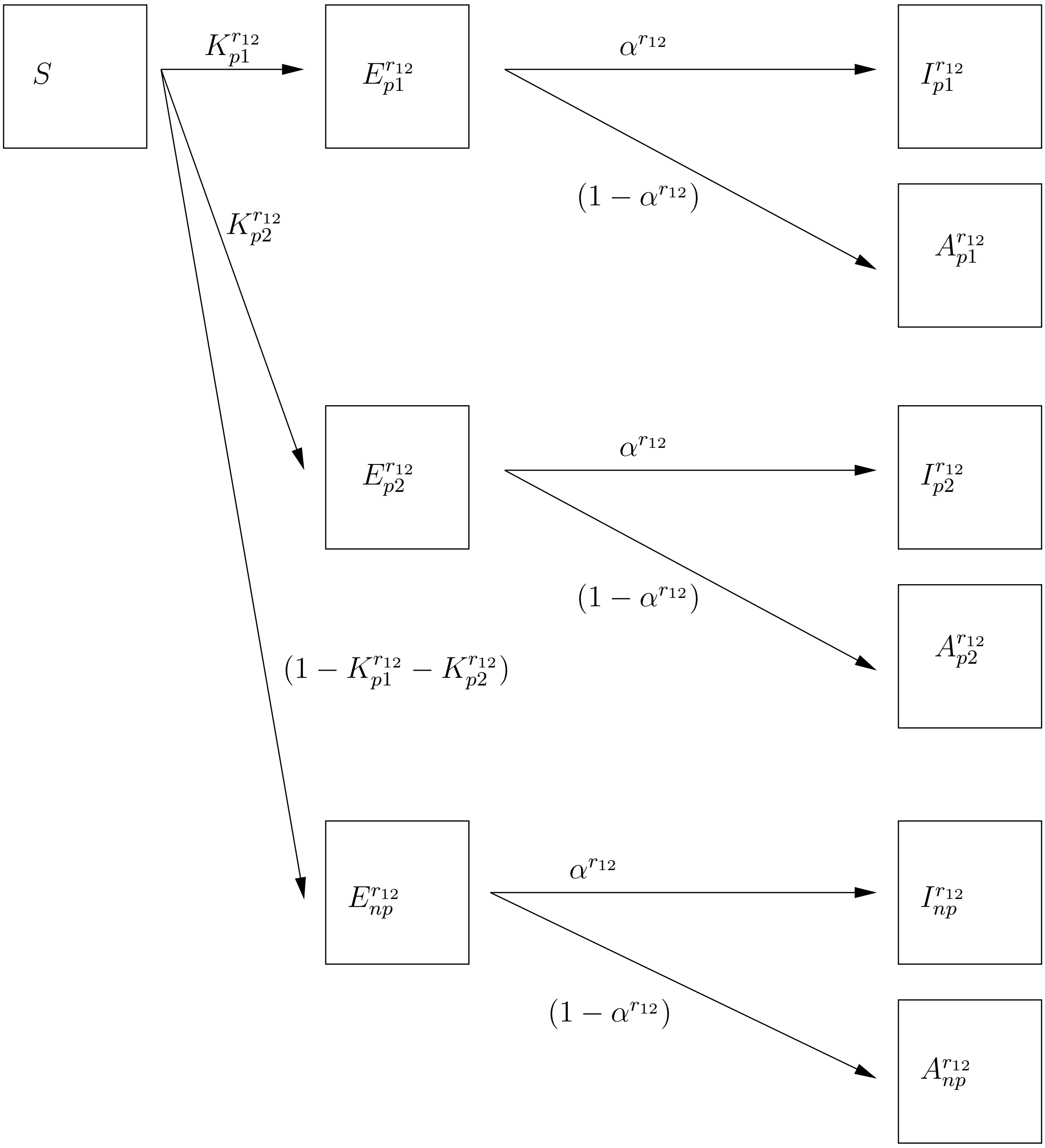

Figure S23: Tree diagram for multi-resistant strain exposures

We have four force-of-infection row vectors (1×30):

$$\vec{\lambda^{w}}' = \beta \begin{bmatrix} e_{i1} \\ e_{i2} \\ e_{t1} \\ e_{t2} \\ 1 \\ 0 \\ 0 \\ 0 \\ 0 \\ 0 \\ 0 \\ 0 \\ 0 \\ 0 \\ 0 \\ 0 \\ 0 \\ 0 \\ e_{i1}\chi \\ e_{i2}\chi \\ \chi \\ 0 \\ 0 \\ 0 \\ 0 \\ 0 \\ 0 \\ 0 \\ 0 \\ 0 \end{bmatrix}, \vec{\lambda^{r_1}}' = \beta\phi^{r_1} \begin{bmatrix} 0 \\ 0 \\ 0 \\ 0 \\ 0 \\ 1 \\ e_{i2} \\ 1 \\ e_{t2} \\ 1 \\ 0 \\ 0 \\ 0 \\ 0 \\ 0 \\ 0 \\ 0 \\ 0 \\ 0 \\ 0 \\ 0 \\ \chi \\ e_{i2}\chi \\ \chi \\ 0 \\ 0 \\ 0 \\ 0 \\ 0 \\ 0 \end{bmatrix}, \vec{\lambda^{r_2}}' = \beta\phi^{r_2} \begin{bmatrix} 0 \\ 0 \\ 0 \\ 0 \\ 0 \\ 0 \\ 0 \\ 0 \\ 0 \\ 0 \\ e_{i1} \\ 1 \\ e_{t1} \\ 1 \\ 1 \\ 0 \\ 0 \\ 0 \\ 0 \\ 0 \\ 0 \\ 0 \\ 0 \\ 0 \\ e_{i1}\chi \\ \chi \\ \chi \\ 0 \\ 0 \\ 0 \end{bmatrix}, \vec{\lambda^{r_{12}}}' = \beta\phi^{r_{12}} \begin{bmatrix} 0 \\ 0 \\ 0 \\ 0 \\ 0 \\ 0 \\ 0 \\ 0 \\ 0 \\ 0 \\ 0 \\ 0 \\ 0 \\ 0 \\ 0 \\ 1 \\ 1 \\ 1 \\ 0 \\ 0 \\ 0 \\ 0 \\ 0 \\ 0 \\ 0 \\ 0 \\ 0 \\ \chi \\ \chi \\ \chi \end{bmatrix} \tag{S164}$$

We construct column vectors $\vec{F^w}$, $\vec{F^{r_1}}$, $\vec{F^{r_2}}$ and $\vec{F^{r_{12}}}$ as follows:

$$\vec{F^w} = \begin{bmatrix} \alpha_{p1}^{w}\left(1-\rho_{p1}\right)e_{s1}K_{p1}^{w} \\ \alpha_{p2}^{w}\left(1-\rho_{p2}\right)e_{s2}K_{p2}^{w} \\ \alpha_{np}^{w}\left(1-\rho_{t1}\right)f_1\psi\left(1-K_{p1}^{w}-K_{p2}^{w}\right) \\ \alpha_{np}^{w}\left(1-\rho_{t2}\right)f_2\psi\left(1-K_{p1}^{w}-K_{p2}^{w}\right) \\ \alpha_{np}^{w}\left(1-\psi\right)\left(1-K_{p1}^{w}-K_{p2}^{w}\right) \\ \alpha_{p1}^{r_1}\rho_{p1}e_{s1}K_{p1}^{w} \\ 0 \\ \alpha_{np}^{w}\rho_{t1}f_1\psi\left(1-K_{p1}^{w}-K_{p2}^{w}\right) \\ 0 \\ 0 \\ 0 \\ \alpha_{p2}^{r_2}\rho_{p2}e_{s2}K_{p2}^{w} \\ 0 \\ \alpha_{np}^{w}\rho_{t2}f_2\psi\left(1-K_{p1}^{w}-K_{p2}^{w}\right) \\ 0 \\ 0 \\ 0 \\ 0 \\ \left(1-\alpha_{p1}^{w}\right)\left(1-\rho_{p1}\right)e_{s1}K_{p1}^{w} \\ \left(1-\alpha_{p2}^{w}\right)\left(1-\rho_{p2}\right)e_{s2}K_{p2}^{w} \\ \left(1-\alpha_{np}^{w}\right)\left(1-K_{p1}^{w}-K_{p2}^{w}\right) \\ \left(1-\alpha_{p1}^{r_1}\right)\rho_{p1}e_{s1}K_{p1}^{w} \\ 0 \\ 0 \\ 0 \\ \left(1-\alpha_{p2}^{r_2}\right)\rho_{p2}e_{s2}K_{p2}^{w} \\ 0 \\ 0 \\ 0 \\ 0 \end{bmatrix}, \vec{F^{r_1}} = \begin{bmatrix} 0 \\ 0 \\ 0 \\ 0 \\ 0 \\ \alpha_{p1}^{r_1}K_{p1}^{r_1} \\ \alpha_{p2}^{r_1}\left(1-\rho_{p2}\right)e_{s2}K_{p2}^{r_1} \\ \alpha_{np}^{r_1}f_1\psi\left(1-K_{p1}^{r_1}-K_{p2}^{r_1}\right) \\ \alpha_{np}^{r_1}\left(1-\rho_{t2}\right)f_2\psi\left(1-K_{p1}^{r_1}-K_{p2}^{r_1}\right) \\ \alpha_{np}^{r_1}\left(1-\psi\right)\left(1-K_{p1}^{r_1}-K_{p2}^{r_1}\right) \\ 0 \\ 0 \\ 0 \\ 0 \\ 0 \\ 0 \\ \alpha^{r_{12}}\rho_{p2}e_{s2}K_{p2}^{r_1} \\ \alpha_{np}^{r_1}\rho_{t2}f_2\psi\left(1-K_{p1}^{r_1}-K_{p2}^{r_1}\right) \\ 0 \\ 0 \\ 0 \\ \left(1-\alpha_{p1}^{r_1}\right)K_{p1}^{r_1} \\ \left(1-\alpha_{p2}^{r_1}\right)\left(1-\rho_{p2}\right)e_{s2}K_{p2}^{r_1} \\ \left(1-\alpha_{np}^{r_1}\right)\left(1-K_{p1}^{r_1}-K_{p2}^{r_1}\right) \\ 0 \\ 0 \\ 0 \\ 0 \\ \left(1-\alpha^{r_{12}}\right)\rho_{p2}e_{s2}K_{p2}^{r_1} \\ 0 \end{bmatrix} \tag{S165}$$

and

$$\vec{F}^{r_2} = \begin{bmatrix} 0 \\ 0 \\ 0 \\ 0 \\ 0 \\ 0 \\ 0 \\ 0 \\ 0 \\ 0 \\ \alpha_{p1}^{r_2}\left(1-\rho_{p1}\right)e_{s1}K_{p1}^{r_2} \\ \alpha_{p2}^{r_2}K_{p2}^{r_2} \\ \alpha_{np}^{r_2}\left(1-\rho_{t1}\right)f_1\psi\left(1-K_{p1}^{r_2}-K_{p2}^{r_2}\right) \\ \alpha_{np}^{r_2}f_2\psi\left(1-K_{p1}^{r_2}-K_{p2}^{r_2}\right) \\ \alpha_{np}^{r_2}\left(1-\psi\right)\left(1-K_{p1}^{r_2}-K_{p2}^{r_2}\right) \\ \alpha^{r_{12}}\rho_{p1}e_{s1}K_{p1}^{r_2} \\ 0 \\ \alpha_{np}^{r_2}\rho_{t1}f_1\psi\left(1-K_{p1}^{r_2}-K_{p2}^{r_2}\right) \\ 0 \\ 0 \\ 0 \\ 0 \\ 0 \\ 0 \\ \left(1-\alpha_{p1}^{r_2}\right)\left(1-\rho_{p1}\right)e_{s1}K_{p1}^{r_2} \\ \left(1-\alpha_{p2}^{r_2}\right)K_{p2}^{r_2} \\ \left(1-\alpha_{np}^{r_2}\right)\left(1-K_{p1}^{r_2}-K_{p2}^{r_2}\right) \\ \left(1-\alpha^{r_{12}}\right)\rho_{p1}e_{s1}K_{p1}^{r_2} \\ 0 \\ 0 \end{bmatrix}, \vec{F}^{r_{12}} = \begin{bmatrix} 0 \\ 0 \\ 0 \\ 0 \\ 0 \\ 0 \\ 0 \\ 0 \\ 0 \\ 0 \\ 0 \\ 0 \\ 0 \\ 0 \\ 0 \\ \alpha^{r_{12}}K_{p1}^{r_{12}} \\ \alpha^{r_{12}}K_{p2}^{r_{12}} \\ \alpha^{r_{12}}\left(1-K_{p1}^{r_{12}}-K_{p2}^{r_{12}}\right) \\ 0 \\ 0 \\ 0 \\ 0 \\ 0 \\ 0 \\ 0 \\ 0 \\ 0 \\ \left(1-\alpha^{r_{12}}\right)K_{p1}^{r_{12}} \\ \left(1-\alpha^{r_{12}}\right)K_{p2}^{r_{12}} \\ \left(1-\alpha^{r_{12}}\right)\left(1-K_{p1}^{r_{12}}-K_{p2}^{r_{12}}\right) \end{bmatrix} \tag{S166}$$

R_0 is the maximum eigenvalue of a 30×30 matrix. As in the single-drug model we can put the matrix into block-triangular form where each block is constructed from $\vec{F}^x$ and $\vec{\lambda}^x$ ($x \in$

$\{w, r_1, r_2, r_{12}\}$). There are four non-zero eigenvalues:

$$R_0 = \frac{\beta}{\gamma} \times \max \begin{cases} \left(\alpha_{p1}^{w} + \left(1-\alpha_{p1}^{w}\right)\chi\right)\left(1-\rho_{p1}\right) e_{s1} e_{i1} K_{p1}^{w} \\ \qquad + \left(\alpha_{p2}^{w} + \left(1-\alpha_{p2}^{w}\right)\chi\right)\left(1-\rho_{p2}\right) e_{s2} e_{i2} K_{p2}^{w} \\ \qquad + \left(\alpha_{np}^{w}\left[e_{t1} f_1 \left(1-\rho_{t1}\right)\psi + e_{t2} f_2 \left(1-\rho_{t2}\right)\psi + (1-\psi)\right]\right. \\ \qquad\qquad \left. + \left(1-\alpha_{np}^{w}\right)\chi\right)\left(1-K_{p1}^{w}-K_{p2}^{w}\right), \\ \phi^{r_1}\left\{\left(\alpha_{p1}^{r_1} + \left(1-\alpha_{p1}^{r_1}\right)\chi\right) K_{p1}^{r_1}\right. \\ \qquad + \left(\alpha_{p2}^{r_1} + \left(1-\alpha_{p2}^{r_1}\right)\chi\right)\left(1-\rho_{p2}\right) e_{s2} e_{i2} K_{p2}^{r_1} \\ \qquad + \left(\alpha_{np}^{r_1}\left[f_1 \psi + e_{t2} f_2 \left(1-\rho_{t2}\right)\psi + (1-\psi)\right]\right. \\ \qquad\qquad \left.\left. + \left(1-\alpha_{np}^{r_1}\right)\chi\right)\left(1-K_{p1}^{r_1}-K_{p2}^{r_1}\right)\right\}, \\ \phi^{r_2}\left\{\left(\alpha_{p1}^{r_2} + \left(1-\alpha_{p1}^{r_2}\right)\chi\right)\left(1-\rho_{p1}\right) e_{s1} e_{i1} K_{p1}^{r_2}\right. \\ \qquad + \left(\alpha_{p2}^{r_2} + \left(1-\alpha_{p2}^{r_2}\right)\chi\right) K_{p2}^{r_2} \\ \qquad + \left(\alpha_{np}^{r_2}\left[e_{t1} f_1 \left(1-\rho_{t1}\right)\psi + f_2 \psi + (1-\psi)\right]\right. \\ \qquad\qquad \left.\left. + \left(1-\alpha_{np}^{r_2}\right)\chi\right)\left(1-K_{p1}^{r_2}-K_{p2}^{r_2}\right)\right\}, \\ \phi^{r_{12}}\left\{\alpha^{r_{12}} + \left(1-\alpha^{r_{12}}\right)\chi\right\} \end{cases} \tag{S167}$$

A.2 Treatment and Prophylaxis model

The possible flows are shown in the following figures.

We define the force of infection row vectors (1×18) (note the transpose):

$$\vec{\lambda^{w}}' = \beta \begin{bmatrix} e_i \\ e_t \\ 1 \\ 0 \\ 0 \\ 0 \\ 0 \\ 0 \\ 0 \\ 0 \\ e_i\chi \\ \chi \\ 0 \\ 0 \\ 0 \\ 0 \\ 0 \\ 0 \end{bmatrix}, \vec{\lambda^{r_p}}' = \beta\phi^{r_p} \begin{bmatrix} 0 \\ 0 \\ 0 \\ 1 \\ e_t \\ 1 \\ 0 \\ 0 \\ 0 \\ 0 \\ 0 \\ 0 \\ \chi \\ \chi \\ 0 \\ 0 \\ 0 \\ 0 \end{bmatrix}, \vec{\lambda^{r_t}}' = \beta\phi^{r_t} \begin{bmatrix} 0 \\ 0 \\ 0 \\ 0 \\ 0 \\ 0 \\ e_i \\ 1 \\ 0 \\ 0 \\ 0 \\ 0 \\ 0 \\ 0 \\ e_i\chi \\ \chi \\ 0 \\ 0 \end{bmatrix}, \vec{\lambda^{r_{tp}}}' = \beta\phi^{r_{tp}} \begin{bmatrix} 0 \\ 0 \\ 0 \\ 0 \\ 0 \\ 0 \\ 0 \\ 0 \\ 1 \\ 1 \\ 0 \\ 0 \\ 0 \\ 0 \\ 0 \\ 0 \\ \chi \\ \chi \end{bmatrix} \tag{S168}$$

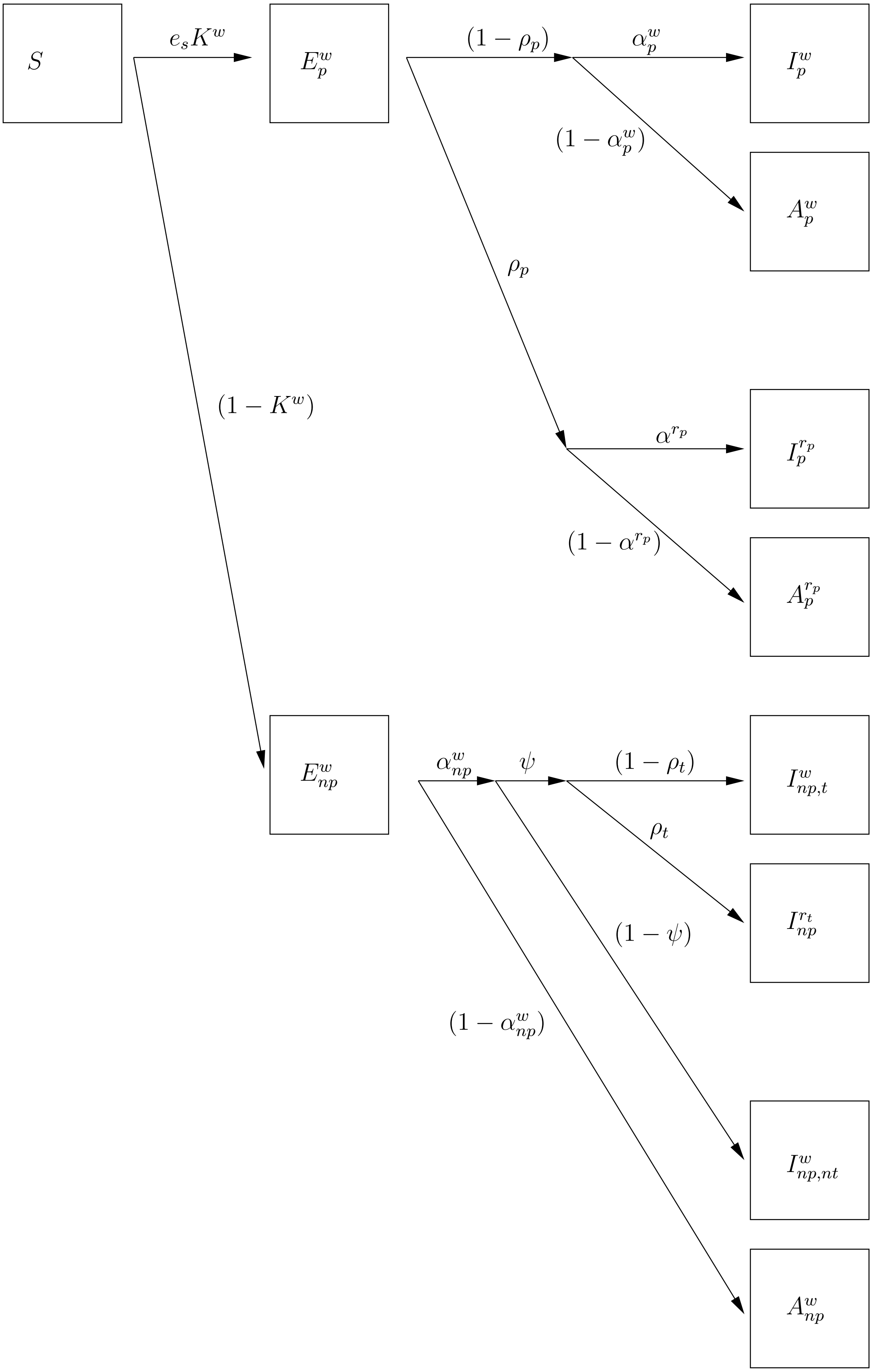

Figure S24: Tree diagram for wild strain exposures

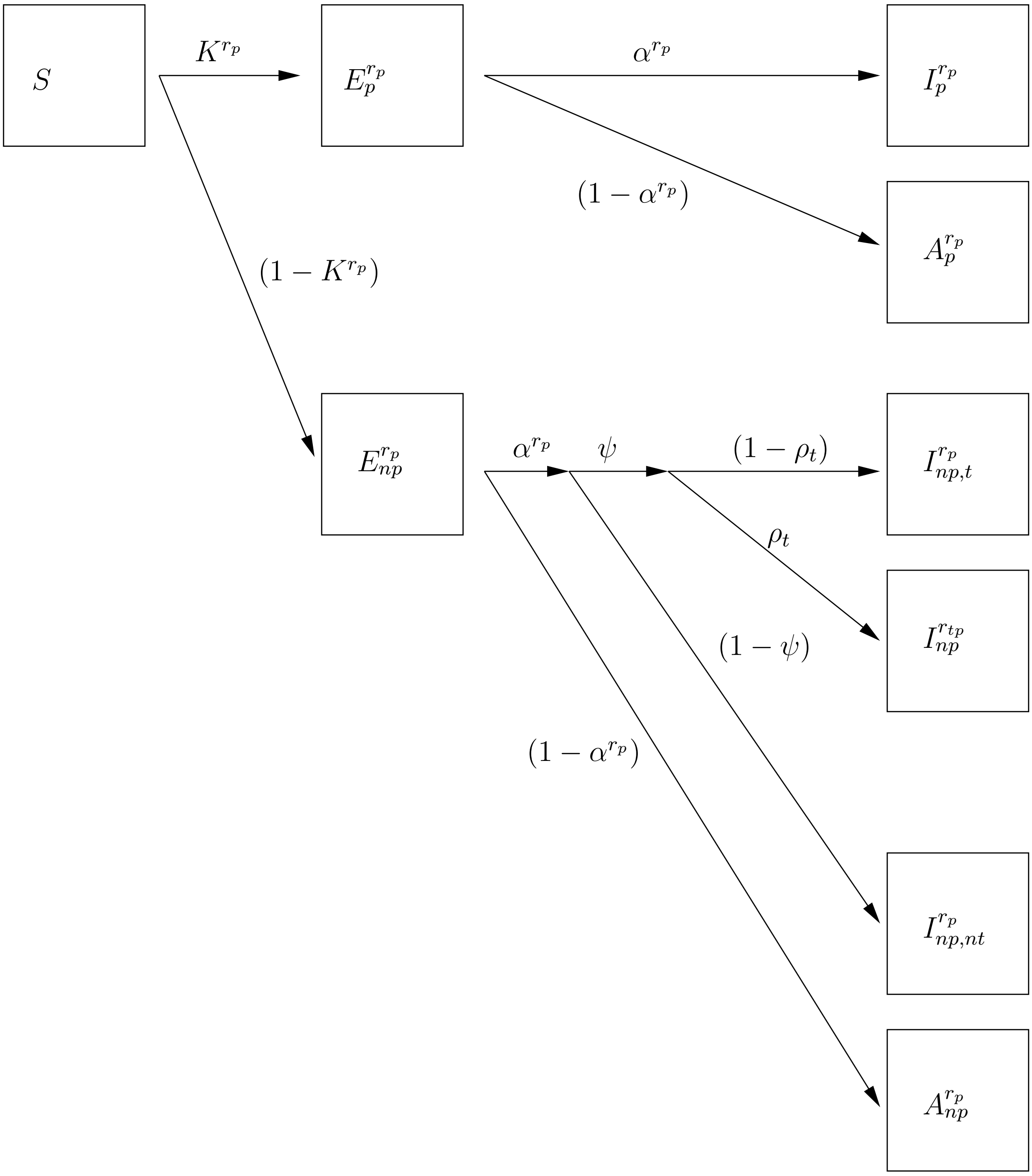

Figure S25: Tree diagram for prophylaxis-resistant strain exposures

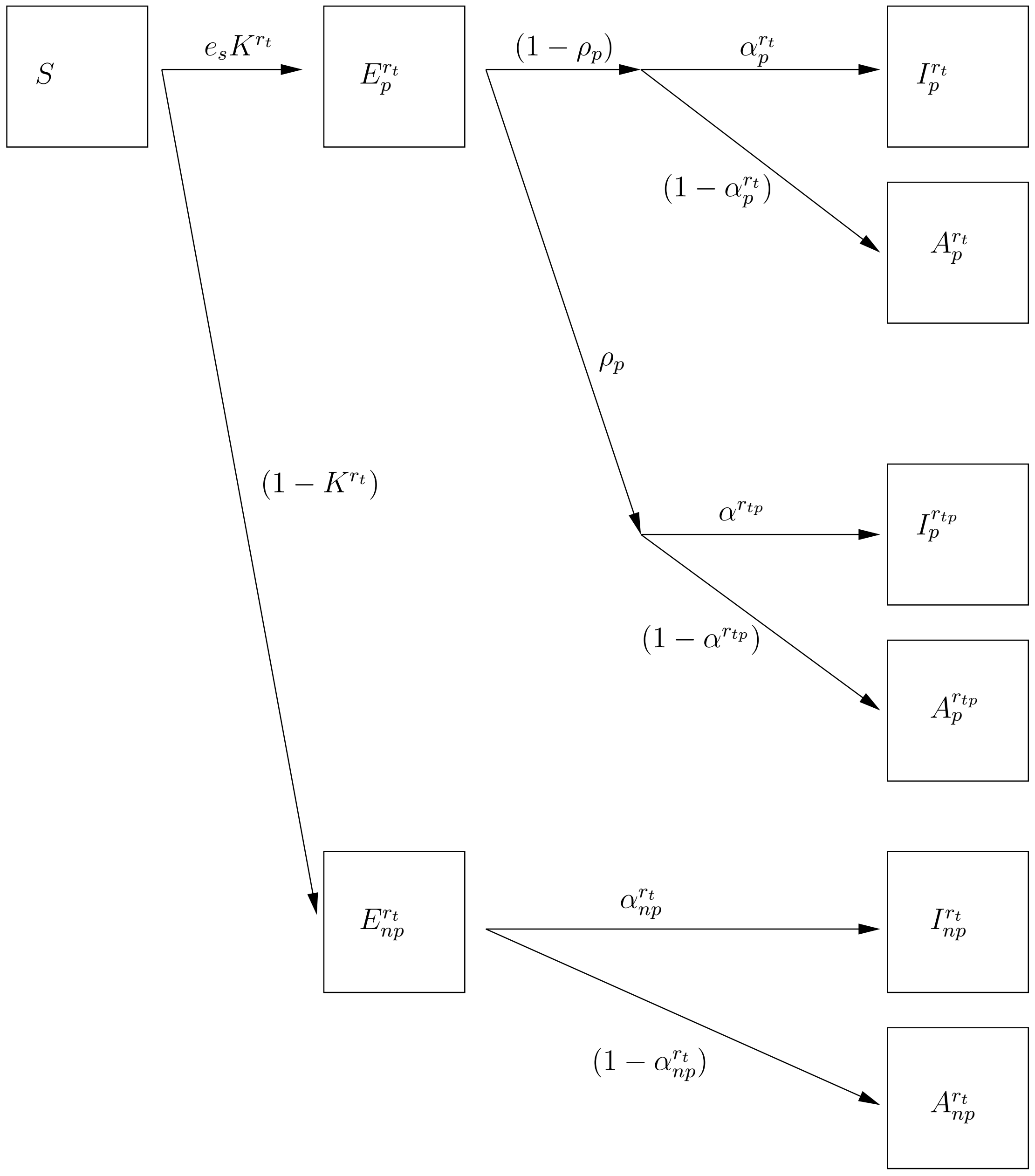

Figure S26: Tree diagram for treatment-resistant strain exposures

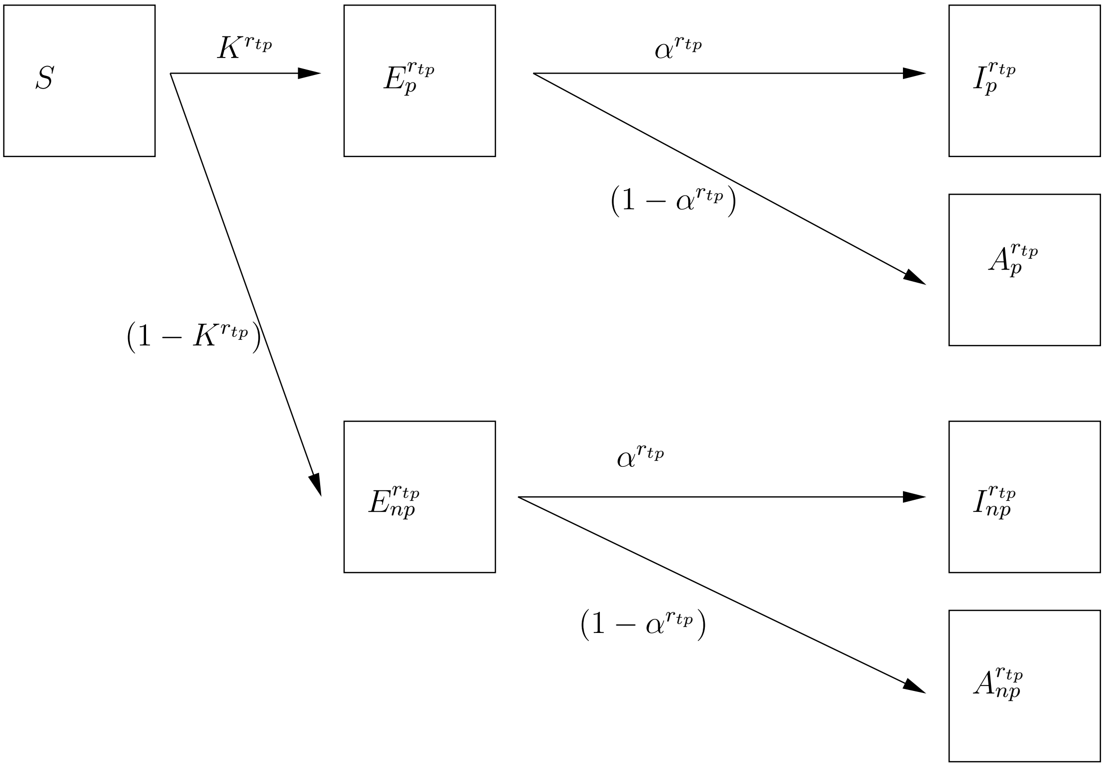

Figure S27: Tree diagram for multi-resistant strain exposures

We construct column vectors $\vec{F^w}$, $\vec{F^{r_p}}$, $\vec{F^{r_t}}$ and $\vec{F^{r_{tp}}}$ as follows:

$$\vec{F^w} = \begin{bmatrix} \alpha_p^w (1-\rho_p) e_s K^w \\ \alpha_{np}^w (1-\rho_t) \psi (1-K^w) \\ \alpha_{np}^w (1-\psi)(1-K^w) \\ \alpha^{r_p} \rho_p e_s K^w \\ 0 \\ 0 \\ 0 \\ \alpha_{np}^w \rho_t \psi (1-K^w) \\ 0 \\ 0 \\ \left(1-\alpha_p^w\right)(1-\rho_p) e_s K^w \\ \left(1-\alpha_{np}^w\right)(1-K^w) \\ (1-\alpha^{r_p}) \rho_p e_s K^w \\ 0 \\ 0 \\ 0 \\ 0 \\ 0 \end{bmatrix}, \vec{F^{r_p}} = \begin{bmatrix} 0 \\ 0 \\ 0 \\ \alpha^{r_p} K^{r_p} \\ \alpha^{r_p} (1-\rho_t) \psi (1-K^{r_p}) \\ \alpha^{r_p} (1-\psi)(1-K^{r_p}) \\ 0 \\ 0 \\ 0 \\ \alpha^{r_p} \rho_t \psi (1-K^{r_p}) \\ 0 \\ 0 \\ (1-\alpha^{r_p}) K^{r_p} \\ (1-\alpha^{r_p})(1-K^{r_p}) \\ 0 \\ 0 \\ 0 \\ 0 \end{bmatrix} \tag{S169}$$

and

$$\vec{F^{r_t}} = \begin{bmatrix} 0 \\ 0 \\ 0 \\ 0 \\ 0 \\ 0 \\ \alpha_p^{r_t}\left(1-\rho_p\right)e_sK^{r_t} \\ \alpha_{np}^{r_t}\left(1-K^{r_t}\right) \\ \alpha^{r_{tp}}\rho_p e_s K^{r_t} \\ 0 \\ 0 \\ 0 \\ 0 \\ 0 \\ \left(1-\alpha_p^{r_t}\right)\left(1-\rho_p\right)e_sK^{r_t} \\ \left(1-\alpha_{np}^{r_t}\right)\left(1-K^{r_t}\right) \\ \left(1-\alpha^{r_{tp}}\right)\rho_p e_s K^{r_t} \\ 0 \end{bmatrix}, \vec{F^{r_{tp}}} = \begin{bmatrix} 0 \\ 0 \\ 0 \\ 0 \\ 0 \\ 0 \\ 0 \\ 0 \\ \alpha^{r_{tp}}K^{r_{tp}} \\ \alpha_{r_{tp}}\left(1-K^{r_{tp}}\right) \\ 0 \\ 0 \\ 0 \\ 0 \\ 0 \\ 0 \\ \left(1-\alpha^{r_{tp}}\right)K^{r_{tp}} \\ \left(1-\alpha^{r_{tp}}\right)\left(1-K^{r_{tp}}\right) \end{bmatrix} \quad \text{(S170)}$$

where K^w, K^{r_p}, K^{r_t} and $K^{r_{tp}}$ are the steady state proportions of $C_p^x/\left(C_p^x + C_{np}^x\right)$ $(x \in \{w, r_p, r_t, r_{tp}\})$.

R_0 is the maximum eigenvalue of the 18×18 matrix $\left(\vec{F^w}\vec{\lambda^w} + \vec{F^{r_p}}\vec{\lambda^{r_p}} + \vec{F^{r_t}}\vec{\lambda^{r_t}} + \vec{F^{r_{tp}}}\vec{\lambda^{r_{tp}}}\right)/\gamma$. We put the matrix into block-triangular form and obtain four non-zero eigenvalues:

$$R_0 = \frac{\beta}{\gamma} \times \max \begin{cases} \left(\alpha_p^w + \left(1-\alpha_p^w\right)\chi\right)\left(1-\rho_p\right)e_se_iK^w \\ \qquad + \left(\alpha_{np}^w\left[e_t\left(1-\rho_t\right)\psi + \left(1-\psi\right)\right] + \left(1-\alpha_{np}^w\right)\chi\right)\left(1-K^w\right), \\ \phi^{r_p}\left\{\alpha^{r_p}\left(K^{r_p} + \left[e_t\left(1-\rho_t\right)\psi + \left(1-\psi\right)\right]\left(1-K^{r_p}\right)\right) + \left(1-\alpha^{r_p}\right)\chi\right\}, \\ \phi^{r_t}\left\{\left(\alpha_p^{r_t} + \left(1-\alpha_p^{r_t}\right)\chi\right)\left(1-\rho_p\right)e_se_iK^{r_t} \right. \\ \qquad \left. + \left(\alpha_{np}^{r_t} + \left(1-\alpha_{np}^{r_t}\right)\chi\right)\left(1-K^{r_t}\right)\right\}, \\ \phi^{r_{tp}}\left\{\alpha^{r_{tp}} + \left(1-\alpha^{r_{tp}}\right)\chi\right\} \end{cases} \quad \text{(S171)}$$

References

J. M. McCaw and J. McVernon. Prophylaxis or treatment? Optimal use of an antiviral stockpile during an influenza pandemic. *Mathematical BioSciences*, 209(2):336–360, 2007. 2, 28
